# Preventing MALT1-mediated CYLD cleavage induces intestinal dysbiosis and reduces EAE severity

**DOI:** 10.1038/s44319-026-00814-4

**Published:** 2026-06-01

**Authors:** Ioannis Skordos, Elisabeth Gilis, Chris Callewaert, Aigerim Aidarova, Mira Haegman, Yasmine Driege, Marja Kreike, Inna S Afonina, Jens Staal, Annelies Demeyer, Dirk Elewaut, Rudi Beyaert

**Affiliations:** 1 Unit of Molecular Signal Transduction in Inflammation, VIB Center for Inflammation Research, VIB, Ghent, Belgium; 2 Ghent University, Department of Biomedical Molecular Biology, Ghent, Belgium; 3 Molecular Immunology and Inflammation Unit, VIB Center for Inflammation Research, VIB, Ghent, Belgium; 4 Ghent University, Department of Rheumatology, Department of Internal Medicine and Pediatrics, Ghent, Belgium; 5 Ghent University, Center for Microbial Ecology and Technology, Ghent, Belgium; 6 Present Address: Ghent University, Department of Head and Skin, Ghent, Belgium

**Keywords:** Immunology, Molecular Biology of Disease, Post-translational Modifications & Proteolysis

## Abstract

The paracaspase MALT1 is essential for lymphocyte activation and also plays roles in non-immune cells and cancer. Its protease activity regulates immune signaling by cleaving specific substrates, making it a promising therapeutic target. However, broad inhibition of MALT1 protease activity causes multiorgan inflammation in mice, highlighting the need to understand the effects of individual substrate cleavage. We generated CYLD(R321A) knock-in mice expressing a MALT1-resistant form of the deubiquitinase CYLD. These mice are healthy, with normal lymphocyte development and preserved immune signaling. Unlike MALT1 protease-dead mice, they do not develop spontaneous inflammation. Notably, they exhibit altered gut microbiota and reduced disease severity in a model of multiple sclerosis. Together, our work shows that blocking cleavage of a single MALT1 substrate is sufficient to modulate microbiota and neuroinflammation without causing overt defects in lymphocyte cell development or activation, providing in vivo evidence for substrate-specific targeting of MALT1 as a refined therapeutic strategy.

## Synopsis

Specific inhibition of MALT1-mediated CYLD cleavage in mice changes gut microbiota and reduces experimentally induced neuroinflammation without impacting lymphocyte development or activation, providing evidence for substrate-specific targeting of MALT1 as a refined therapeutic strategy.

- Lymphocytes from MALT1 cleavage-resistant CYLD(R321A) mice exhibit normal NF-κB and MAPK activation.
- Preventing MALT1-mediated CYLD cleavage has no effect on T or B cell development and homeostasis.
- Inhibition of MALT1-mediated CYLD cleavage reduces EAE without affecting T cell priming.
- Protection against EAE is lost upon co housing CYLD(R321A) and wild type mice in the same cage.
- Expression of cleavage-resistant CYLD leads to intestinal dysbiosis, maybe contributing to altered EAE susceptibility.

## Abstract (web summary)

Specific inhibition of MALT1-mediated CYLD cleavage in mice changes gut microbiota and reduces experimentally induced neuroinflammation without impacting lymphocyte development or activation, providing evidence for substrate-specific targeting of MALT1 as a refined therapeutic strategy.

## Graphical abstract

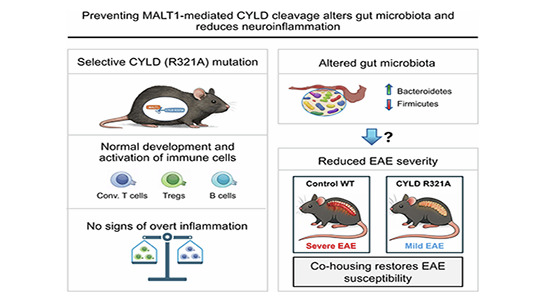

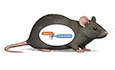

## Introduction

The paracaspase “mucosa-associated lymphoid tissue lymphoma translocation protein 1” (MALT1) is a central regulator of immune responses and plays important roles in health and disease. MALT1 is broadly expressed across hematopoietic and non-hematopoietic tissues, where it mediates signaling downstream of various receptors, including the T cell receptor (TCR), B cell receptor (BCR), C-type lectin receptors, and G protein-coupled receptors (Ruland and Hartjes, 2019). Additionally, gain-of-function mutations in upstream signaling components can enhance MALT1 signaling (Juilland and Thome, 2016). Hyperactivation of MALT1 has been implicated in several inflammatory disorders and malignancies, particularly lymphomas (Gomez Solsona et al, 2022). Conversely, MALT1 deficiency results in immunodeficiency due to impaired antigen-induced T and B cell activation, as well as defective development of T regulatory cells (Treg), marginal zone B cells, and peritoneal B1 B cells (Ruefli-Brasse et al, 2003; Ruland et al, 2003). MALT1 knock-out (KO) mice also fail to form germinal centers and exhibit impaired humoral responses to blood-borne antigens (Ruland et al, 2003; Bornancin et al, 2015; Lee et al, 2017; Yu et al, 2015). With age, these mice develop atopic-like dermatitis, preceded by Th2 skewing and elevated serum IgE levels (Demeyer et al, 2019a). Collectively, these findings underscore the essential role of MALT1 in maintaining immune homeostasis.

MALT1 activation occurs through the assembly of the CARD-CC/BCL10/MALT1 (CBM) signalosome, which includes one of the “caspase activation and recruitment domain and a coiled-coil domain” (CARD-CC) family proteins (CARD-9, -10, -11, -14), “B-cell CLL/lymphoma 10” (BCL10), and MALT1 itself. This complex promotes MALT1 oligomerization and activation (Ruland and Hartjes, 2019). Within this complex, MALT1 functions both as a scaffold that recruits downstream signaling molecules and as an arginine-specific cysteine protease. Through these dual roles, MALT1 activates IκB kinase and c-Jun N-terminal kinase (JNK), leading to the induction of nuclear factor-ĸB (NF-κB) and activator protein-1 (AP-1) transcription factors, respectively (Ruland and Hartjes, 2019). In addition to its scaffolding function, MALT1 cleaves a range of substrates to regulate both transcription-dependent and -independent immune responses (Ruland and Hartjes, 2019).

The importance of MALT1 proteolytic activity is highlighted by studies showing that its inhibition blocks antigen receptor-induced T cell activation in vitro (Biswas et al, 2022; Bardet et al, 2018). Small-molecule MALT1 inhibitors have shown therapeutic potential in preclinical models of multiple sclerosis (Mc Guire et al, 2014; Biswas et al, 2021), psoriasis (Nunettsu Asaba et al, 2023; Van Nuffel et al, 2020), and colitis (Liu et al, 2016), as well as in cancers characterized by constitutive MALT1 activation (Mempel and Krappmann, 2022). Several MALT1 inhibitors are currently in clinical trials for B-cell malignancies (Hamp et al, 2021). However, long-term inhibition of MALT1 protease activity has been associated with systemic autoimmunity, driven by impaired Treg cell function and increased effector T cell activity (Biswas et al, 2022; Martin et al, 2020; Demeyer et al, 2020; Dumont et al, 2020). Unlike MALT1 KO mice, MALT1 protease-dead (MALT1-PD) knock-in (KI) mice develop a fatal autoinflammatory syndrome (Bornancin et al, 2015; Yu et al, 2015; Gewies et al, 2014; Jaworski et al, 2014), which has been attributed to a Treg-intrinsic defect (Cheng et al, 2019; Rosenbaum et al, 2019). These findings underscore the critical role of MALT1 in immune regulation. However, the specific MALT1 substrates responsible for the autoimmune phenotype in MALT1-PD mice remain unidentified. MALT1 substrates include regulators of NF-ĸB or JNK signaling (e.g., A20, cylindromatosis (CYLD), RelB, HOIL-1), mRNA stability (e.g., Regnase-1, Roquin-1, Roquin-2, and N4BP1), and cell adhesion or cytoskeletal dynamics (e.g., BCL10, LIMA1, and Tensin-3) (Moud et al, 2024). Additional substrates have recently been identified through bioinformatics approaches (Bell et al, 2022). Previous studies have shown that mice expressing MALT1-insensitive HOIL-1, Roquin-1, or Tensin-3 maintain normal immune homeostasis under steady-state conditions (Schmidt et al, 2023; Juilland et al, 2023; Skordos et al, 2023). Thus, the substrate(s) responsible for the severe autoimmune phenotype in MALT1-PD mice remain elusive.

In this study, we focus on the MALT1 substrate CYLD (Staal et al, 2011), a deubiquitinase that negatively regulates NF-κB and JNK signaling (Sun, 2010). Previous studies showed that CYLD-deficient mice exhibit multiple immune abnormalities, including immune cell development (Reiley et al, 2007; Jin et al, 2007; Reiley et al, 2006) and Treg function (Lee et al, 2021; Reissig et al, 2012). Human CYLD is cleaved by MALT1 at arginine R324 in response to TCR (Staal et al, 2011) and BCR (Minderman et al, 2023) triggering, generating an N-terminal 40 kDa fragment containing two cytoskeleton-associated protein glycine-rich (CAP-Gly) domains and a C-terminal 70 kDa fragment containing a third CAP-Gly domain and the catalytic ubiquitin-specific protease (USP) domain (Staal et al, 2011). Overexpression of non-cleavable CYLD in cell lines has suggested a role for CYLD cleavage in modulating NF-ĸB and JNK signaling (Staal et al, 2011; Minderman et al, 2023), but the physiological relevance of these findings remains unclear, especially since primary T cells from MALT1-PD mice show no defects in these pathways upon TCR stimulation (Bornancin et al, 2015; Jaworski et al, 2014; Gewies et al, 2014). Here, we describe the generation of CYLD(R321A) KI mice expressing a MALT1 cleavage-resistant form of mouse CYLD. Using this model, we investigate the functional role of CYLD cleavage in immune cell development and function under both homeostatic and inflammatory conditions.

## Results

### Lymphocytes from CYLD(R321A) mice exhibit normal NF-κB and MAPK activation

**Figure EV1:**
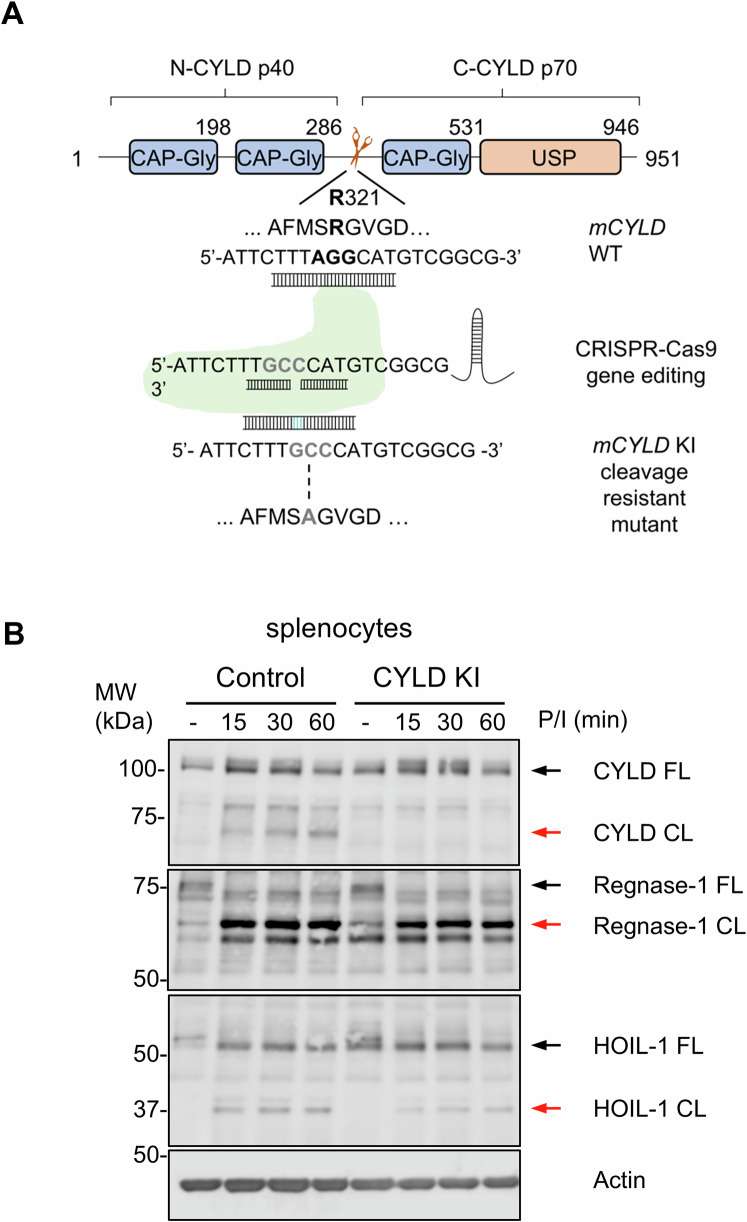
Inhibition of CYLD cleavage in CYLD(R321A) splenocytes. (**A**) Schematic representation of the MALT1 cleavage site at R321 in mouse CYLD, leading to an N-terminal fragment (p40) and a C-terminal fragment (p70). CRISPR/Cas9 gene editing was used to generate non-cleavable CYLD(R321A) KI mice. (**B**) Immunoblot analysis of MALT1-mediated cleavage of CYLD, Regnase-1, and HOIL-1 in WT and CYLD KI splenocytes stimulated with PMA plus ionomycin (P/I) for the indicated times. Splenocytes from three mice per genotype (8–12 weeks old) were pooled. Full-length (FL) and cleaved (CL) forms of CYLD (p70), Regnase-1 (p55), and HOIL-1 (p22) are indicated by black and red arrowheads, respectively. Actin served as a loading control. All proteins shown, including the loading control, were run on the same gel. Data were representative of two experiments with similar results. Source data are available online for this figure.

*Cyld(R321A)* KI mice, referred to hereafter as CYLD KI, were generated using CRISPR/Cas9 genome editing to introduce a single amino acid substitution (R321A), replacing the critical arginine at the MALT1 cleavage site with alanine (corresponding to R324 in human CYLD), (Fig. EV1A). Both homozygous and heterozygous CYLD KI mice were born at expected Mendelian ratios (1:2:1) and remained healthy and macroscopically normal for at least 12 months, similar to wild-type (WT) controls. We confirmed that CYLD cleavage was effectively blocked in splenocytes from CYLD KI mice following stimulation with phorbol 12-myristate 13-acetate plus ionomycin (P/I), which mimics antigen-receptor signaling (Fig. EV1B). Notably, cleavage of other substrates, including Regnase-1 and HOIL-1, remained intact, indicating that MALT1 protease activity was preserved.

**Figure 1:**
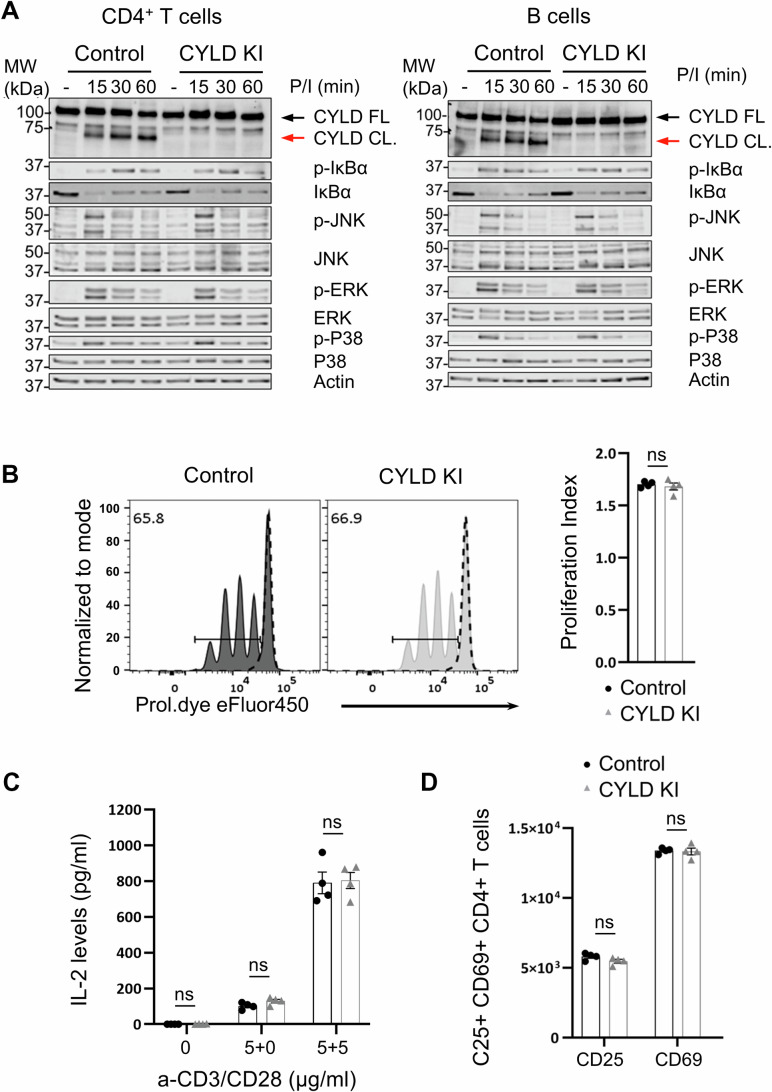
In vitro activation of T and B cells from CYLD KI mice. (**A**) NF-κB and MAPK signaling. Purified splenic CD4^+^ T and B cells from control WT and CYLD KI mice (4–5 mice per group) were stimulated with PMA plus ionomycin (P/I) for the indicated times. Cell extracts were analyzed by immunoblotting for phosphorylation of IκBα, JNK, ERK, and p38 MAPK. Actin served as a loading control. All proteins shown, including the loading control, were run on the same gel. Full-length (FL) and cleaved (CL) forms of CYLD (p70) are indicated by black and red arrowheads, respectively. (**B**) T cell proliferation. Splenic CD4^+^ T cells from control WT and CYLD KI mice were labeled with the proliferation dye eFluor450 and stimulated in vitro with anti-CD3/CD28 for 72 h. (Left panel) Representative flow cytometry plots showing the percentage of proliferating CD4^+^ T cells; dashed lines indicate non-stimulated controls. (Right panel) Quantification of proliferating CD4^+^ T cells. (**C**) IL-2 production. ELISA quantification of IL-2 in supernatants of splenic CD4^+^ T cells stimulated for 24 h with 5 μg/mL anti-CD3 with or without 5 μg/mL anti-CD28. (**D**) Activation marker expression. Mean fluorescence intensity of CD25 and CD69 on CD25^+^CD69^+^CD4^+^ T cells 72 h post anti-CD3/CD28 stimulation. Statistical analysis (**B**–**D**) was performed using a two-tailed unpaired Student’s *t*-test; ns non-significant. (**B**–**D**) Bar graphs show means ± SEM, with data points representing biological replicates (individual mice). Sample sizes: (**B**–**D**) *n* = 4 (WT) and *n* = 4 (CYLD KI) per group. All mice were 10–12 weeks old. All experiments were performed twice (**A**,** C**,** D**) and three times (**B**) with similar results. Source data are available online. Source data are available online for this figure.

Given that overexpression of cleavage-resistant CYLD(R324A) in Jurkat T cells has been reported to impair JNK activation and reduce interleukin (IL)-2 production following TCR stimulation (Staal et al, 2011), we investigated whether endogenous CYLD cleavage affects signaling in primary lymphocytes. Purified splenic CD4^+^ T cells and B cells from CYLD KI and WT mice were stimulated with P/I. In contrast to previous findings in Jurkat T cells, JNK phosphorylation was comparable between WT and CYLD KI lymphocytes (Fig. 1A). Similarly, phosphorylation of ERK and p38 mitogen-activated protein kinases (MAPKs), as well as phosphorylation and degradation of inhibitor of κBα (IκBα), a hallmark of canonical NF-κB activation, were unaffected by the R321A mutation. Functional assays further revealed no significant differences in proliferation, IL-2 production, or expression of activation markers CD25 and CD69 in CD4^+^ T cells stimulated with anti-CD3/CD28 (Fig. 1B–D). Together, these results demonstrate that MALT1-mediated cleavage of CYLD is not required for NF-κB or MAPK signaling, nor for key aspects of lymphocyte activation.

### Preventing MALT1-mediated CYLD cleavage does not affect T cell development or homeostasis

**Figure 2:**
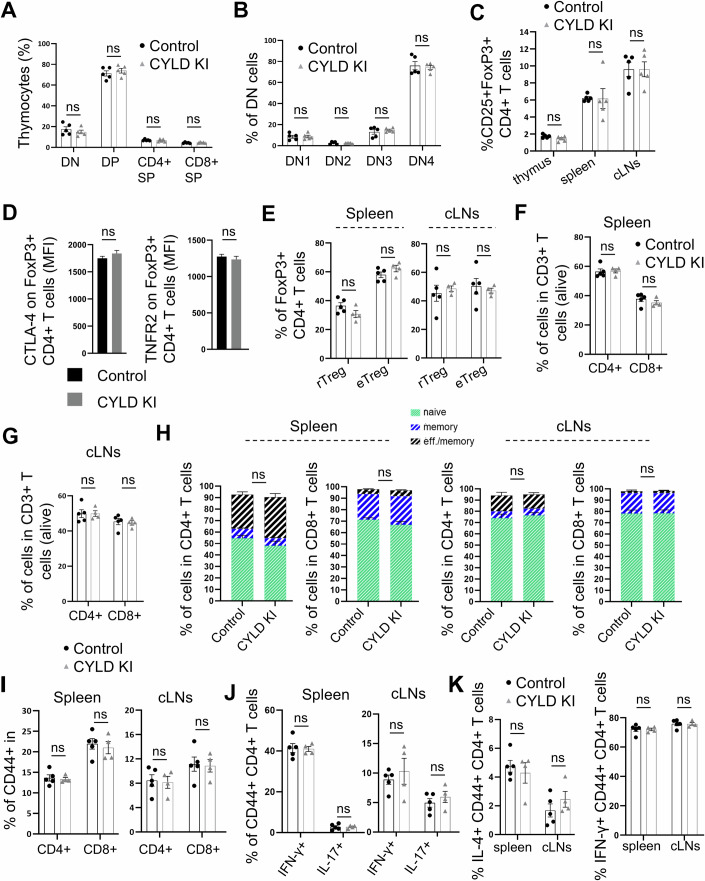
T cell development, activation and subsets in CYLD KI mice. (**A**) Frequencies of thymocyte subsets in CYLD KI and control WT mice (8–12 weeks old). Populations include double-negative (DN; CD4^−^CD8^−^CD3^−^), double-positive (DP; CD4^+^CD8^+^CD3^−^), and single-positive (SP; CD4^+^CD3^−^ or CD8^+^CD3^−^) cells. (**B**) Frequencies of DN thymocyte subsets: DN1 (CD44^+^CD25^−^), DN2 (CD44^+^CD25^+^), DN3 (CD44^−^CD25^+^), and DN4 (CD44^−^CD25^−^). (**C**) Frequency of Tregs (CD25^+^FoxP3^+^) gated on CD3^−^CD4^+^CD8^−^ T cells in the thymus, and on CD3^+^CD4^+^CD8^−^ T cells in the spleen and cervical lymph nodes (cLNs). (**D**) Mean fluorescence intensity of CTLA-4 and TNFR2 on splenic FoxP3^+^CD4^+^ Tregs. (**E**) Frequencies of resting (naïve) Tregs (rTreg; CD62L^+^CD44^−^) and effector Tregs (eTreg; CD62L^−^CD44^+^) within the FoxP3^+^CD4^+^ Treg population in the spleen and cLNs. (**F**,** G**) Frequencies of CD4^+^ and CD8^+^ T cells within the CD3^+^ T cell population in the spleen and cLNs. (**H**) Frequencies of naïve (CD62L^+^CD44^−^), central memory (CD62L^+^CD44^+^) and effector/memory (CD62L^−^CD44^+^) CD4^+^ and CD8^+^ T cell subsets in the spleen and cLNs. (**I**) Percentage of CD44-expressing CD4^+^ and CD8^+^ T cells isolated from spleen and cLNs of WT control and CYLD KI mice. (**J**) Quantification of IFN-γ^+^ and IL-17^+^ CD44^+^CD4^+^ T cells from spleen and cLNs. (**K**) Frequencies of IL-4^+^ CD44^+^CD4^+^ T cells (left panel) and IFN-γ^+^ CD44^+^CD8^+^ T cells (right panel) from spleen and cLNs. Cells were stimulated in vitro for 4 h with PMA plus ionomycin (P/I) in the presence of brefeldin A. All bar graphs show means ± SEM, with individual data points representing single animals (biological replicates). Comparisons between two groups were performed using a two-tailed unpaired Student’s *t*-test; ns non-significant. Sample sizes: (**A**–**C**) *n* = 5 (WT) and *n* = 5 (CYLD KI); (**D**) *n* = 5 (WT) and *n* = 6 (CYLD KI); (**E**–**H**) *n* = 5 (WT) and *n* = 4 (CYLD KI); (**I**–**K**) *n* = 5 (WT) and *n* = 4 (CYLD KI). All mice were 8–14 weeks old. The data were representative of at least three experiments with similar results. Source data are available online. Source data are available online for this figure.

**Figure EV2:**
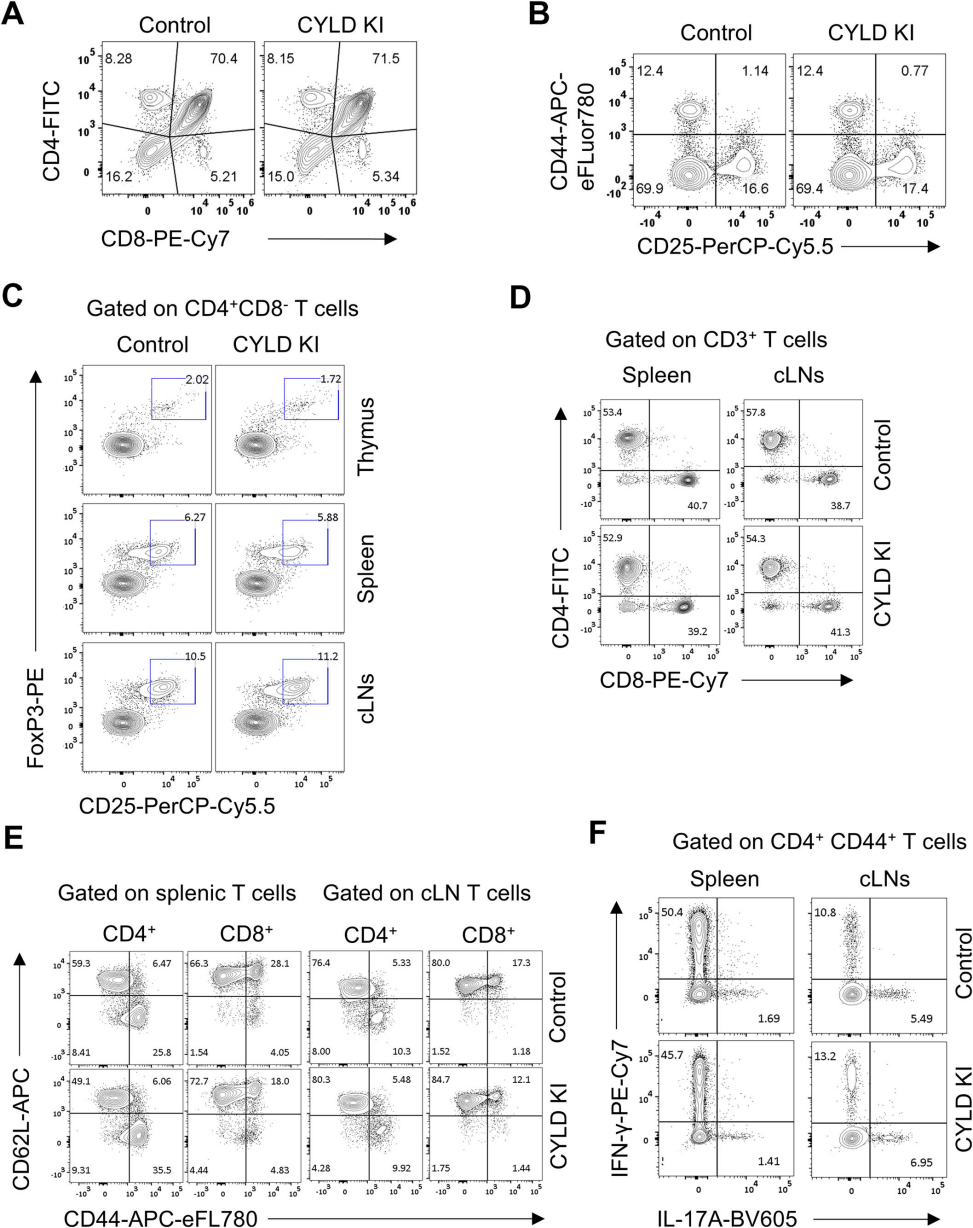
Normal thymic and peripheral T cell compartments in CYLD KI mice. (**A**) Representative contour plots showing the frequencies of thymocyte subsets in CYLD KI and control WT mice (8–12 weeks old). Populations include double-negative (DN; CD4^−^CD8^−^CD3^−^), double-positive (DP; CD4^+^CD8^+^CD3^−^), and single-positive (SP; CD4^+^CD3^-^ or CD8^+^CD3^−^) cells. (**B**) Representative contour plots showing the distribution of DN thymocyte subsets: DN1 (CD44^+^CD25^-^), DN2 (CD44^+^CD25^+^), DN3 (CD44^−^CD25^+^), and DN4 (CD44^−^CD25^−^). (**C**) Representative contour plots showing the frequency of Tregs (CD25^+^FoxP3^+^) gated on CD3^-^CD4^+^CD8^-^ T cells in the thymus, and on CD3^+^CD4^+^CD8^-^ T cells in the spleen and cervical lymph nodes (cLNs). (**D**) Representative contour plots showing the frequencies of CD4^+^ and CD8^+^ T cells within the CD3^+^ T cell population in the spleen and cLNs. (**E**) Representative contour plots showing the frequencies of naïve (CD62L^+^CD44^−^), central memory (CD62L^+^CD44^+^) and effector/memory (CD62L^−^CD44^+^) CD4^+^ and CD8^+^ T cell subsets in the spleen and cLNs. (**F**) Representative contour plots of IFN-γ^+^ and IL-17^+^ CD44^+^CD4^+^ T cells from spleen and cLNs. Numbers within gates indicate the percentage of cells in each population. Data are representative of at least three experiments with similar results.

MALT1-PD mice exhibit defects in early thymocyte differentiation, although conventional T cell levels in secondary lymphoid tissues remain unaffected (Bornancin et al, 2015; Gewies et al, 2014; Jaworski et al, 2014). To determine whether CYLD cleavage contributes to these processes, we analysed thymocyte populations in CYLD KI mice. Flow cytometric analysis revealed normal distributions of CD4^−^CD8^−^ double-negative (DN), CD4^+^CD8^+^ double-positive (DP), CD4^+^CD8^−^ single-positive (CD4^+^ SP) and CD4^−^CD8^+^ SP (CD8^+^ SP) thymocytes (Figs. 2A and EV2A). Additionally, the proportions of DN1 (CD44^+^CD25^−^), DN2 (CD44^+^CD25^+^), DN3 (CD44^−^CD25^+^), and DN4 (CD44^−^CD25) subsets were comparable to WT controls, indicating intact early thymocyte differentiation in CYLD KI mice (Figs. 2B and EV2B). These findings suggest that MALT1-mediated CYLD cleavage is dispensable for both early and late stages of thymocyte maturation.

The severe autoimmunity observed in MALT1-PD mice has been attributed to impaired development and function of thymic Treg (FoxP3^+^CD25^+^CD4^+^), leading to uncontrolled activation of conventional T cells (Jaworski et al, 2014; Rosenbaum et al, 2019; Bornancin et al, 2015; Gewies et al, 2014; Yu et al, 2015; Demeyer et al, 2019b; Cheng et al, 2019). Given CYLD’s role in regulating Treg cell plasticity (Reiley et al, 2007; Reissig et al, 2012; Lee et al, 2021), we investigated whether preventing CYLD cleavage affects Treg development. Flow cytometry revealed that Treg frequencies in the thymus, spleen, and cervical lymph nodes were similar between WT and CYLD KI mice (Figs. 2C and EV2C). Furthermore, expression levels of the immunosuppressive markers CTLA-4 and TNFR2 both reduced in MALT1-PD Tregs (Rosenbaum et al, 2019; Demeyer et al, 2019b), were unchanged in CYLD KI mice, as measured by mean fluorescence intensity in splenic FoxP3^+^CD4^+^ T cells (Fig. 2D). The conversion of resting Tregs (rTreg; CD62L^+^CD44^−^) to effector Tregs (eTreg; CD62L^−^CD44^+^), a process regulated by MALT1 signaling (Rosenbaum et al, 2019), was also unaffected (Fig. 2E). These results indicate that CYLD cleavage is not required for Treg development, stability, or suppressive function. Consistent with normal thymocyte development, CYLD KI mice displayed comparable proportions of CD4^+^ and CD8^+^ T cells in peripheral lymphoid organs, including spleen and cervical lymph nodes (Figs. 2F,G and EV2D). The distribution of naïve, memory, and effector/memory subsets among CD4^+^ and CD8^+^ T cells was also similar between genotypes (Figs. 2H and EV2E).

Upon in vitro stimulation with P/I for 4 h, CD44 expression, a marker of T cell activation, was equivalent in CD4^+^ and CD8^+^ T cells from CYLD KI and control mice (Fig. 2I). Importantly, cytokine production by activated T cells was unaffected. Levels of interferon (IFN)-γ-producing (Th1) and IL-17-producing (Th17) CD44^+^CD4^+^ T cells were comparable between CYLD KI and WT mice in both spleen and cervical lymph nodes (Figs. 2J and EV2F). This contrasts with MALT1-PD mice, which exhibit increased Th1 responses and impaired Th17 differentiation (Jaworski et al, 2014; Gewies et al, 2014; Bornancin et al, 2015; Demeyer et al, 2019b). Similarly, IL-4-producing (Th2) CD44^+^CD4^+^ T cells and IFN-γ-producing CD44^+^CD8^+^ T cells were present at normal levels in CYLD KI mice (Fig. 2K). Collectively, these findings demonstrate that expression of the MALT1 cleavage-resistant CYLD(R321A) mutant does not disrupt T cell development, activation, or differentiation.

### B cell development and function are unaffected in CYLD KI mice

**Figure 3:**
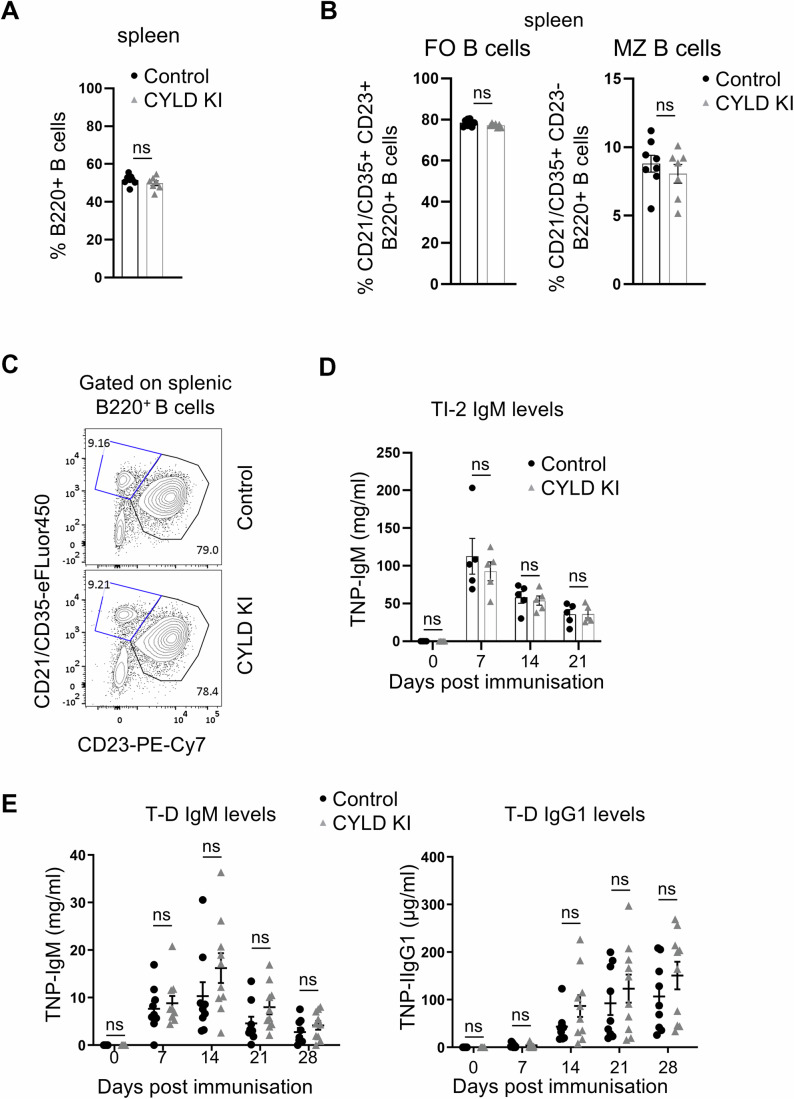
B cell subsets and antigen-specific immune responses in CYLD KI mice. (**A**) Frequency of B220^+^ B cells in the spleen of WT control and CYLD KI mice. (**B**) Frequencies of follicular (FO; CD23^+^CD21^+^/CD35^+^) and marginal zone (MZ; CD23^-^CD21^+^/CD35^+^) B cells within the B220^+^ population. (**C**) Representative contour plots show gating strategies for FO (black gate) and MZ (blue gate) B cells. (**D**) T cell-independent type 2 (TI-2) immune response. Mice were immunized with TNP-Ficoll, and serum anti-TNP IgM levels were measured by ELISA on days 0, 7, 14, and 21 post-immunization. (**E**) T cell-dependent (T-D) immune response. Mice were immunized with TNP-KLH in Alum, and serum anti-TNP IgM and IgG1 levels were measured by ELISA on days 0, 7, 14, 21, and 28 post-immunization. All graphs show means ± SEM, with individual data points representing single animals (biological replicates). Statistical analysis: (**A**, **B**) two-tailed unpaired Student’s *t*-test; ns non- significant. (**D**) Two-way ANOVA test with Bonferroni correction; ns non-significant. (**E**) Mann–Whitney *U*-test; ns non-significant. Numbers in contour plots indicate percentages of gated populations. Sample sizes: (**A**,** B**) *n* = 8 (WT) and *n* = 7 (CYLD KI, 8–13 weeks old); (**D**) *n* = 5 (WT) and *n* = 5 (CYLD KI, 8–13 weeks old). (**E**) Data were from two experiments combined, with a total of *n* = 9 (WT) and *n* = 10 (CYLD KI) biological replicates (individual mice, 8–13 weeks old). Data were representative of three (**A**–**C**) and two (**D**) experiments with similar results. Source data are available online. Source data are available online for this figure.

MALT1-PD mice exhibit a marked reduction in splenic marginal zone B cells, while maintaining normal levels of follicular B cells (Gewies et al, 2014; Bornancin et al, 2015; Yu et al, 2015). In contrast, CYLD-deficient mice display an expansion of the marginal zone B cell compartment (Jin et al, 2007). These observations prompted us to explore whether expression of a cleavage-resistant CYLD mutant would alter B cell subset distribution. Flow cytometric analysis revealed that the overall frequency of B220^+^ B cells in the spleen was comparable between CYLD KI and WT mice (Fig. 3A). Moreover, the proportions of marginal zone and follicular B cells were unchanged (Fig. 3B,C), indicating that MALT1-mediated cleavage of CYLD is not required for B cell development or maturation. Given that MALT1-PD mice fail to mount effective humoral responses to both T cell-independent and T cell-dependent antigens (Yu et al, 2015; Bornancin et al, 2015; Gewies et al, 2014; Jaworski et al, 2014), we next assessed whether CYLD cleavage influences antigen-specific antibody production. CYLD KI and WT mice were immunized with the T cell-independent type 2 antigen trinitrophenyl-Ficoll (TNP-Ficoll) and the T cell-dependent antigen trinitrophenyl-keyhole limpet hemocyanin (TNP-KLH). Serum analysis revealed no significant differences in TNP-specific antibody levels between genotypes following immunization with either antigen (Fig. 3D,E). Together, these results demonstrate that preventing CYLD cleavage by MALT1 does not impair B cell development or humoral immune responses.

### CYLD KI mice are hyporesponsive to experimental autoimmune encephalomyelitis

**Figure 4:**
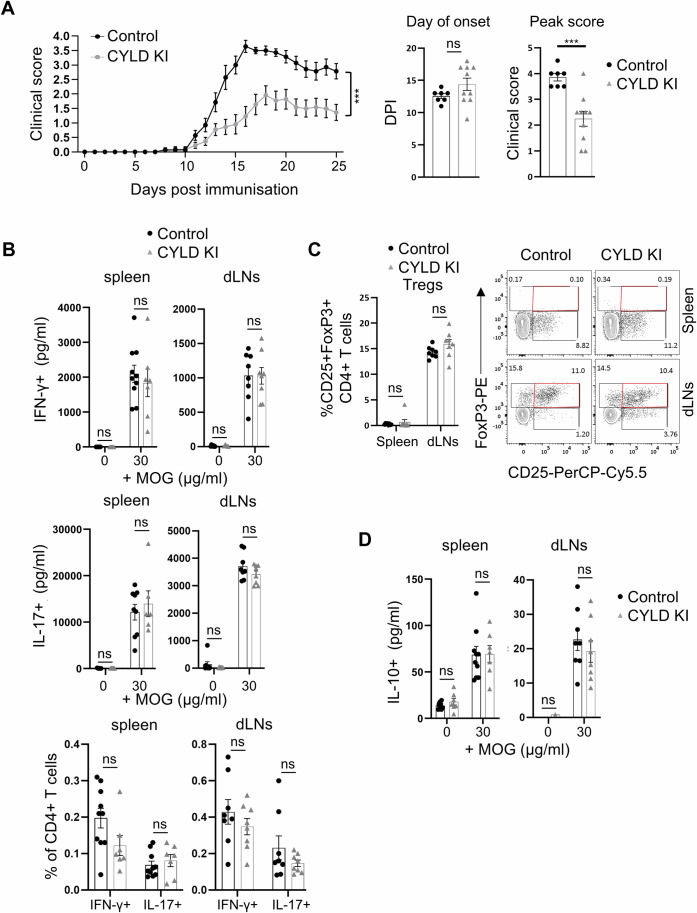
EAE sensitivity of WT and CYLD KI mice. (**A**) Clinical evaluation of EAE in WT control and CYLD KI mice immunized with MOG_33–55_. Shown are daily clinical scores, day of disease onset, and peak clinical scores. (**B**) Antigen-specific T cell responses following MOG_33–45_ immunization. Ten days post-immunization, spleens and draining lymph nodes (dLNs) were harvested, and single-cell suspensions were restimulated in vitro with 30 μg/mL MOG_33–55_. IFN-γ (upper panel) and IL-17 (middle panel) levels in culture supernatants were quantified by Bioplex assay at 48 and 96 h post-restimulation, respectively. Frequencies of IFN-γ and IL-17 expressing CD4^+^ T cells (bottom panel) were analysed by flow cytometry at 72 h post-restimulation. (**C**) Frequencies and representative contour plots of FoxP3^+^ CD25^+^ Tregs within the CD4^+^ T cell population in spleen and dLNs, 10 days post-immunization. (**D**) IL-10 levels in supernatants from MOG_33–55_ restimulated spleen and dLN cell cultures, as described in (b). Statistical analysis: (**A**) EAE clinical score curve: Two-way repeated measures (RM)ANOVA***p = 0.0005). Line graphs show means ± SEM (**A**–**D**). Two-tailed unpaired Student’s *t*-test (for bar graphs in (**A**) ***p = 0.0009; ns non-significant). Bar graphs show means ± SEM, with data points representing biological replicates (individual mice). Sample sizes: (**A**) *n* = 7 (WT) and *n* = 10 (CYLD KI); (**B**) *n* = 10 (WT) and *n* = 7 (CYLD KI); (**C**) *n* = 8 per group; (**D**) *n* = 9 (WT) and *n* = 8 (CYLD KI). All mice were 8–14 weeks old. Experiments were performed two (**C**) and three times (**A**,** B**,** D**) with similar results. Source data are available online. Source data are available online for this figure.

Although CYLD KI mice exhibit normal T or B cell activation under steady-state conditions, this does not preclude a role for MALT1-mediated CYLD cleavage in immune responses under pathological conditions. To explore this, we investigated the involvement of CYLD cleavage in experimental autoimmune encephalomyelitis (EAE), a model of multiple sclerosis in which MALT1 protease inhibition is known to confer protection (Bornancin et al, 2015; Mc Guire et al, 2014; Gewies et al, 2014). EAE was induced in CYLD KI and WT control mice by immunization with myelin oligodendrocyte glycoprotein (MOG)_35–55_ peptide emulsified in complete Freund’s adjuvant (CFA). Disease progression was monitored over 25 days. CYLD KI mice developed significantly milder disease compared to controls, as evidenced by reduced clinical scores (Fig. 4A). While disease onset was similar between groups, CYLD KI mice exhibited lower peak disease severity.

Given that MALT1-PD mice show impaired generation of MOG-specific Th1 and Th17 cells (Jaworski et al, 2014), we hypothesized that reduced EAE severity in CYLD KI mice might result from altered CD4⁺ T cell priming. To test this, splenocytes and draining lymph node cells were harvested 10 days post-immunization and restimulated in vitro with MOG_35–55_. Levels of IFN-γ and IL-17A produced by restimulated cells were comparable between WT and CYLD KI mice (Fig. 4B). Similarly, the frequencies of IFN-γ^+^ and IL-17^+^ MOG-specific CD4^+^ T cells were not significantly different, suggesting that T cell priming was unaffected by the CYLD mutation.

We next examined whether Tregs, which are critical for controlling EAE severity by limiting autoreactive T cell expansion (Kohm et al, 2002; Zhang et al, 2004; Stephens et al, 2009), contributed to the observed phenotype. Flow cytometric analysis revealed similar frequencies of FoxP3⁺ Tregs in the spleen and draining lymph nodes of CYLD KI and WT mice at day 10 post-immunization (Fig. 4C). Additionally, IL-10 secretion by MOG-restimulated cells was unchanged between groups (Fig. 4D), indicating that Treg abundance and function were not responsible for the attenuated EAE in CYLD KI mice.

**Figure EV3:**
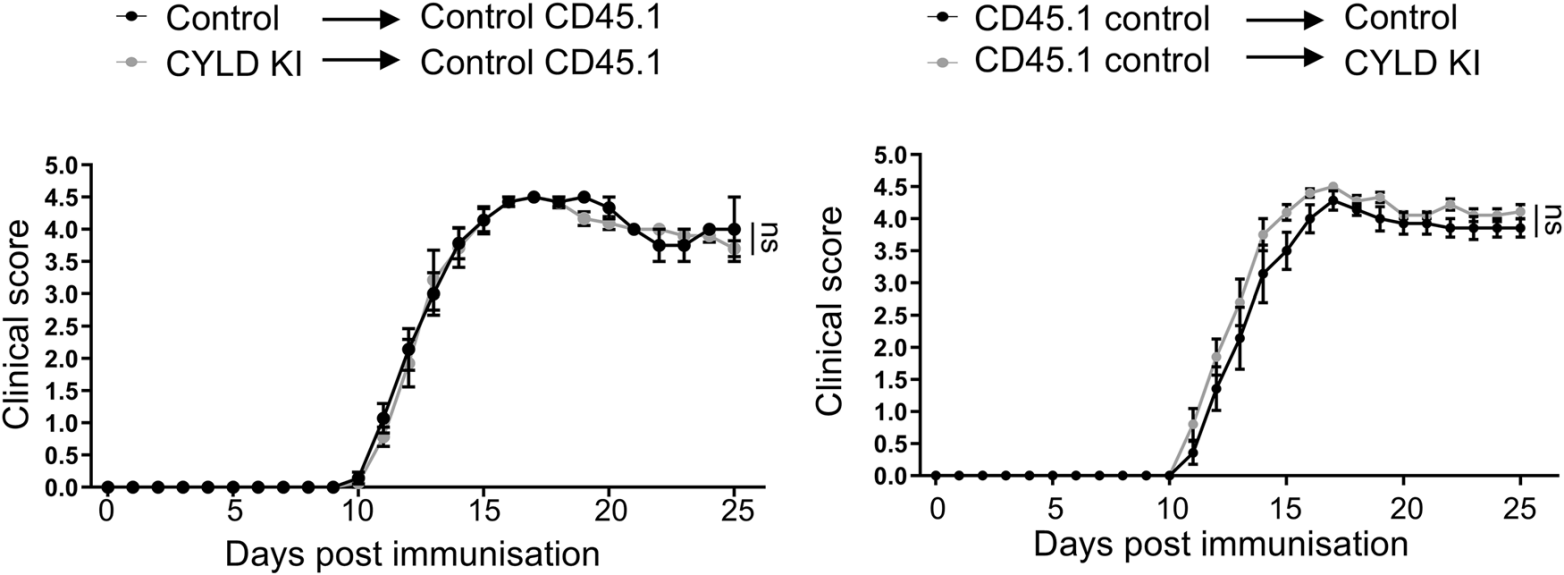
Bone marrow chimera experiments assessing the contribution of hematopoietic and non-hematopoietic compartments to EAE susceptibility. Clinical scores of EAE in bone marrow chimeric mice. Lethally irradiated CD45.1⁺ control (WT) recipient mice were reconstituted with 2 × 10⁶ bone marrow cells from either CD45.2⁺ WT or CD45.2⁺ CYLD KI donors (left panel). In the reciprocal experiment, lethally irradiated CD45.2⁺ WT or CYLD KI recipient mice were reconstituted with CD45.1⁺ WT bone marrow (right panel). After 10 weeks, chimeras were immunized with MOG_35–55_, and disease progression was monitored daily for 25 days. Statistical analysis: Two-way repeated measures ANOVA. Sample sizes: left panel *n* = 7 mice per group; right panel *n* = 7 WT → WT and 10 WT → CYLD KI mice. All mice were 8–14 weeks old. Data were presented as means ± SEM and are representative of two experiments with similar results. Source data are available online for this figure.

To further dissect the contribution of hematopoietic versus non-hematopoietic compartments, including glial or meningeal antigen-presenting cells, we performed reciprocal bone marrow chimera experiments. Lethally irradiated control (WT) recipient mice were reconstituted with bone marrow from either WT or CYLD KI donors, and conversely, WT or CYLD KI recipient mice were reconstituted with bone marrow from CD45.1⁺ WT donors. Ten weeks after reconstitution, mice were subjected to induction of EAE as described above. In both experimental settings, no significant differences in disease severity were observed between the respective groups (Fig. EV3). These results indicate that expression of cleavage-resistant CYLD in either the hematopoietic or non-hematopoietic compartment alone is insufficient to account for the reduced disease severity. Instead, the protective phenotype may require coordinated contributions from both compartments. Together, these findings support a role for MALT1-mediated CYLD cleavage in modulating neuroinflammation during EAE.

### Hyporesponsiveness of CYLD KI mice to EAE is lost upon co-housing with WT mice

**Figure 5:**
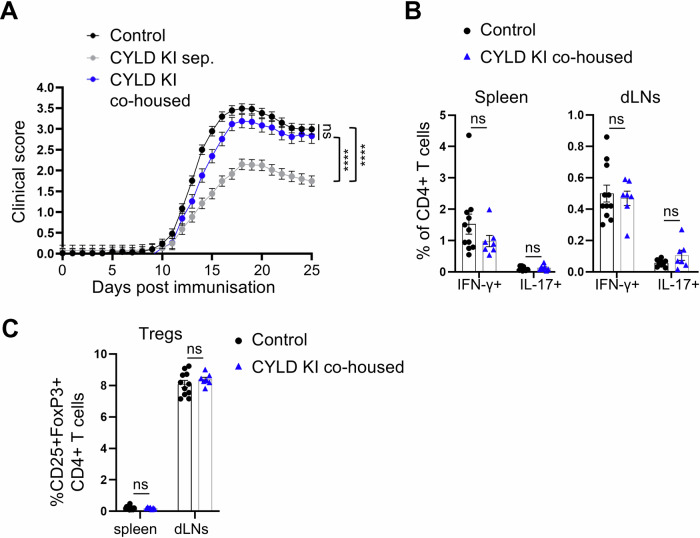
Impact of co-housing on EAE sensitivity in CYLD KI mice. (**A**) EAE disease progression in WT and CYLD KI mice housed either together or in separate cages. Line graphs show means ± SEM. Data were combined from four experiments, with a total of *n* = 39 (WT), *n* = 33 (CYLD KI separately caged), and *n* = 29 (CYLD KI co-housed) biological replicates (individual mice).. Statistical analysis was performed using a modified *F*-test to assess two-way and three-way interactions (****p < 0.0001 (WT vs CYLD KI separately caged, *p* = 9.72 × 10^−^¹⁴; CYLD KI separately caged vs CYLD KI co-housed, *p* = 0.00001322; ns non-significant)). (**B**) Antigen-specific T cell responses following MOG_33–45_ immunization. Ten days post-immunization, spleens and draining lymph nodes (dLNs) were harvested from co-housed WT and CYLD KI mice. Single-cell suspensions were restimulated in vitro with MOG₃₅–₅₅ for 72 h, and CD4⁺ T cells were analysed for IFN-γ and IL-17 expression by flow cytometry. (**C**) Frequencies of FoxP3^+^CD25^+^ Tregs within the CD4^+^ T cell compartment in spleens and dLNs of co-housed control WT and CYLD KI mice, 10 days post-immunization. Bar graphs shown means ± SEM, with individual data points representing single animals (biological replicates). Statistical analysis was performed using a two-tailed unpaired Student’s *t*-test. Sample sizes: *n* = 11 (WT) and *n* = 7 (CYLD KI). All mice were 8–14 weeks old. (**B**,** C**) All experiments were performed two times, showing similar results. Source data are available online. Source data are available online for this figure.

In the active immunization EAE experiments described above, CYLD KI mice were housed in separate cages from control mice. However, when CYLD KI mice were co-housed with WT mice in the same cage, their hyporesponsiveness to EAE was lost, and disease severity increased to levels comparable to those observed in control mice (Fig. 5A). Despite this change in susceptibility, co-housed CYLD KI and control mice exhibited similar frequencies of MOG-specific Th1 and Th17 cells in peripheral lymphoid tissues 10 days post-immunization (Fig. 5B), consistent with observations in separately housed mice. Likewise, the proportions of immunosuppressive Tregs in the spleen and draining lymph nodes were comparable between co-housed CYLD KI and control mice (Fig. 5C). These findings suggest that the increased EAE susceptibility in co-housed CYLD KI mice is not due to enhanced autoreactive T cell responses or diminished Treg populations, but rather implicates environmental influences.

**Figure 6:**
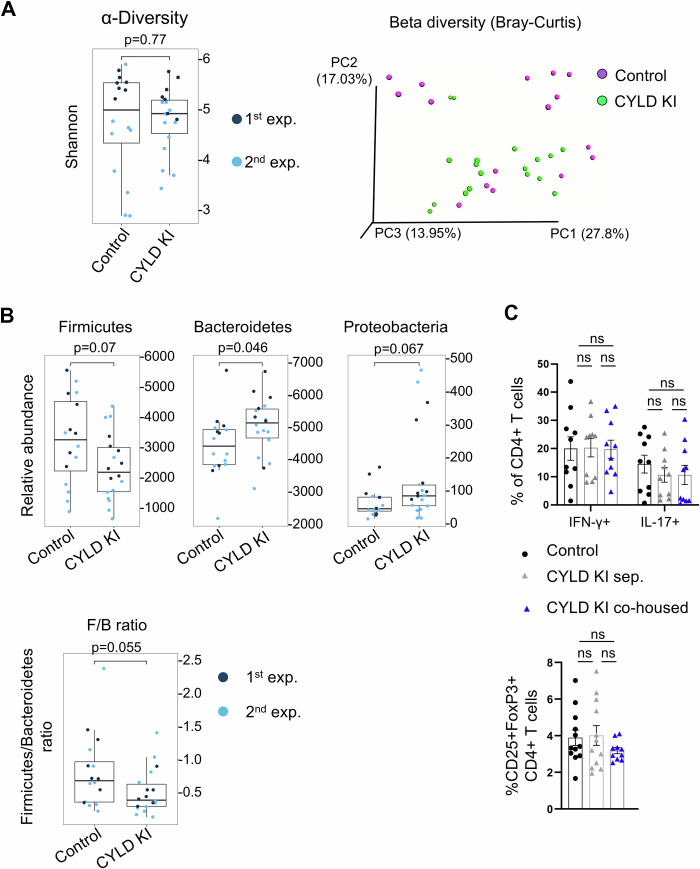
Intestinal microbiome composition in separately caged WT and CYLD KI mice. (**A**) Fecal microbiome analysis of separately housed WT and CYLD KI mice (8–13 weeks old), performed three days prior to MOG_33–55_ immunization. (left panel) Microbial α-diversity is represented by the Shannon index, which accounts for both species richness and evenness. (right panel) Three-dimensional principal coordinate (PC) analysis of Bray–Curtis distances (β-diversity), illustrating microbial community dissimilarity between WT and CYLD KI mice. Data were combined from two experiments; individual data points represent biological replicates (individual mice) from experiment 1 (dark blue) and experiment 2 (light blue); total *n* = 39 WT and 33 CYLD KI mice. Statistical analysis: Mann–Whitney *U*-test; significance threshold *p* < 0.05. (**B**) Relative abundance of major bacterial phyla in fecal samples from (**A**), shown as mean percentages of operational taxonomic units (OTUs). The Firmicutes/Bacteroidetes (F/B) ratio was calculated based on the mean relative abundance of these two phyla. Box plots show median (center line), 25th–75th percentiles (box limits), and 1.5× interquartile range (whiskers); points beyond whiskers are outliers. Statistical analysis: Mann–Whitney *U*-test; significance threshold *P* < 0.05. (**C**) Frequencies of IFN-γ^+^ and IL-17^+^ CD4^+^ T cells (left panel) and CD25^+^ FoxP3^+^ Tregs (right panel) in the small intestinal lamina propria of WT and CYLD KI mice, either separately housed or co-housed. Data were combined from three experiments; *n* = 10 mice (8–13 weeks old) per group. Bar graphs show mean ± SEM, with individual data points representing single animals. Statistical analysis: two-tailed unpaired Student’s *t*-test; ns non-significant. Source data are available online for this figure.

Given the established role of gut microbiota in modulating EAE susceptibility (Regen et al, 2021; Kumar et al, 2016; Berer et al, 2011, 2017), we hypothesized that differences in intestinal microbial composition might underlie the reduced EAE sensitivity observed in CYLD KI mice. To test this, we performed 16S rRNA sequencing on fecal samples collected from separately housed CYLD KI and control mice 3 days prior to EAE induction. While species richness (alpha diversity) was similar between groups, beta-diversity analysis revealed a significant difference in overall microbial composition (*p* = 0.033; Fig. 6A). Notably, CYLD KI mice exhibited a higher relative abundance of Bacteroidetes (*p* = 0.046), with trends towards reduced Firmicutes and increased Proteobacteria (*p* ≤ 0.07; Fig. 6B). Although the Firmicutes/Bacteroidetes (F/B) ratio was only marginally lower in CYLD KI mice (*p* = 0.055), these results suggest that expression of MALT1 cleavage-resistant CYLD leads to gut dysbiosis, which may contribute to altered EAE susceptibility.

**Figure EV4:**
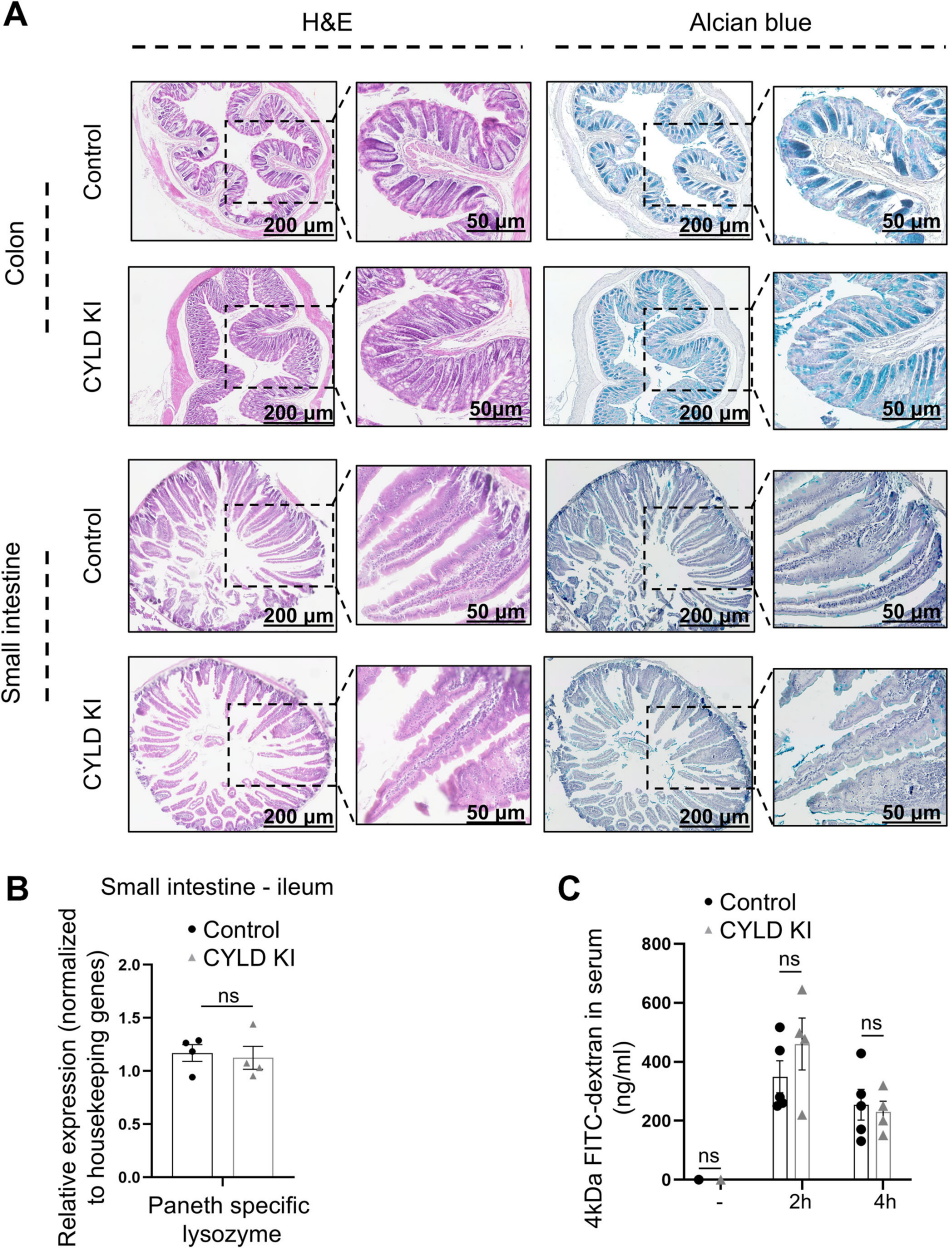
Intestinal morphology and barrier function are preserved in CYLD KI mice. (**A**) Representative histological sections of colon (top) and small intestine (bottom) from naïve-state WT and CYLD KI mice stained with hematoxylin and eosin (H&E, left) or Alcian Blue (right). Scale bar, 200 μm. Insets show higher magnification views. (**B**) Expression levels of the Paneth cell antimicrobial peptide lysozyme in the small intestine of WT and CYLD KI mice, determined by qPCR. (**C**) Intestinal permeability assessed by oral administration of 4-kDa FITC-dextran followed by measurement of serum fluorescence after 2 h (reflecting small intestinal permeability) and 4 h (reflecting colonic permeability). Bar graphs show mean ± SEM, with individual data points representing single animals (biological replicates). Statistical analysis was performed using a two-tailed unpaired Student’s *t*-test (ns non-significant) (**B**) and mixed-effects analysis with Greenhouse–Geisser correction (ns; non-significant) (**C**). Sample sizes: (**A**) *n* = 5 WT and *n* = 5 CYLD KI mice, representative histological sections shown; (**B**) *n* = 4 WT and 4 CYLD KI mice; (**C**) *n* = 5 WT and 4 CYLD KI. All mice were 8–12 weeks old. (**B**,** C**) Data were representative of two experiments with similar results. Source data are available online for this figure.

Because epithelial barrier integrity is a key determinant of host–microbiota interactions, we next examined whether preventing cleavage of CYLD by MALT1 could affect intestinal barrier function. Histological analysis of small intestine and colon sections stained with hematoxylin–eosin and Alcian Blue revealed no detectable differences between WT and CYLD KI mice in tissue architecture or goblet cell distribution (Fig. EV4A). In addition, expression of the Paneth cell antimicrobial peptide lysozyme in the small intestine, assessed by qPCR, was comparable between WT and CYLD KI genotypes (Fig. EV4B). Finally, intestinal permeability was evaluated by oral administration of FITC-dextran (4 kDa), followed by measurement of serum fluorescence after 2 and 4 h, reflecting small intestinal and colonic permeability, respectively. No differences in FITC-dextran translocation were observed between WT and CYLD KI mice (Fig. EV4C). Together, these results indicate that intestinal epithelial morphology, antimicrobial peptide production, and barrier permeability are preserved in CYLD KI mice under steady-state conditions. We next investigated whether alterations in intestinal immune cell populations might contribute to the altered EAE susceptibility of CYLD KI mice. To address this possibility, we analyzed CD4⁺ T cell subsets in the lamina propria of the small intestine. Frequencies of Th1, Th17, and Treg cells were comparable between CYLD KI and control mice, regardless of housing conditions (Fig. 6C). These data indicate that the reduced EAE susceptibility in separately housed CYLD KI mice is not mediated by microbiota-dependent alterations in intestinal CD4^+^ T cell populations, but likely involves other, as yet undefined, mechanisms.

## Discussion

Although CYLD is one of the most frequently studied MALT1 substrates, the physiological relevance of its cleavage by MALT1 remains largely unclear. In this study, we generated and characterized CYLD KI mice expressing a non-cleavable form of CYLD to investigate this question. CYLD is a well-established deubiquitinase that negatively regulates NF-κB signaling in lymphocytes, and its complete deficiency in mice leads to severe multiorgan inflammation due to hyperresponsive T and B cells (Reiley et al, 2007; Hövelmeyer et al, 2007).

Using T and B cells from CYLD KI mice, we demonstrate that MALT1-mediated CYLD cleavage is dispensable for NF-κB, ERK, p38, and JNK signaling following in vitro antigen-receptor stimulation. These findings contradict earlier reports suggesting a role for CYLD cleavage in JNK regulation in Jurkat T cells (Staal et al, 2011), and they also diverge from recent studies implicating CYLD cleavage in promoting NF-κB signaling in BCR-dependent lymphomas (Minderman et al, 2023). These discrepancies may stem from non-physiological protein levels in overexpression systems, which can affect native protein-protein interactions. Importantly, our results align with previous findings in MALT1-PD mice, which showed that MALT1 proteolytic activity is not required for canonical NF-κB or MAPK signaling in antigen-receptor-stimulated lymphocytes (Gewies et al, 2014; Bornancin et al, 2015; Jaworski et al, 2014). To assess the in vivo relevance of CYLD cleavage, we examined T and B cell development and activation in CYLD KI mice. Unlike MALT1-PD mice, which exhibit profound defects in Treg development, CYLD KI mice showed normal Treg maturation in the thymus and normal Treg frequencies in both primary and secondary lymphoid tissues. This observation is consistent with recent studies showing that inhibition of MALT1-mediated cleavage of HOIL-1 or Roquin-1 does not result in a major defect in thymic Treg development (Skordos et al, 2023; Schmidt et al, 2023). The specific MALT1 substrate responsible for the Treg defects in MALT1-PD mice remains unidentified. Regnase-1, a known MALT1 substrate involved in stabilizing *Ctla-4* and *Tnfr2* mRNA (Uehata et al, 2013; Jeltsch et al, 2014), is a strong candidate. Supporting this, transgenic mice expressing a cleavage-resistant Regnase-1 mutant (R111A) exhibit altered T cell function and homeostasis (Kong et al, 2021). However, interpretation of these findings requires caution, as the transgenic was driven by the strong CAG promoter, potentially leading to non-physiological expression levels.

We also found no evidence of effector T cell hyperactivation in CYLD KI mice. The frequencies of Th1, Th2, and Th17 subsets in secondary lymphoid tissues were comparable to those in control mice. Furthermore, the reduced TCR-induced proliferation and IL-2 secretion previously observed in MALT1-PD CD4^+^ T cells cannot be attributed to impaired CYLD cleavage, suggesting the involvement of other MALT1 substrates. Prior work by Baens et al. highlighted the role of MALT1 auto-proteolysis in enabling IL-2 production following TCR activation (Baens et al, 2018). Additionally, *IL-2* mRNA stability—regulated by factors such as Regnase-1 during TCR signaling (Uehata et al, 2013; Jeltsch et al, 2014), may be compromised in MALT1-PD CD4^+^ T cells due to impaired Regnase-1 degradation.

MALT1 protease activity is also essential for marginal zone B cell development, while being dispensable for follicular B cells (Yu et al, 2015; Bornancin et al, 2015; Gewies et al, 2014; Jaworski et al, 2014). Marginal zone B cells are critical for rapid IgM responses to T cell-independent antigens, whereas follicular B cells primarily mediate T cell-dependent responses (Martin et al, 2001). The loss of marginal zone B cells in MALT1-PD mice correlates with their impaired humoral immunity (Yu et al, 2015; Bornancin et al, 2015; Gewies et al, 2014; Jaworski et al, 2014). In contrast, CYLD KI mice exhibited normal development of both B cell subsets and mounted effective immune responses to both T cell-independent and T cell-dependent antigens. These findings indicate that CYLD cleavage is not responsible for the B cell defects observed in MALT1-PD mice.

To investigate the role of CYLD cleavage under inflammatory conditions, we used the EAE model of multiple sclerosis model, in which MALT1-PD mice are known to be protected (Bornancin et al, 2015; Gewies et al, 2014). Similarly, CYLD KI mice exhibited reduced EAE susceptibility compared to WT controls. This protection was not due to impaired T cell priming, as MOG-specific Th1 and Th17 cells were present at similar levels in both groups. Treg frequencies and IL-10 levels in peripheral lymphoid tissues were also comparable. To further determine whether hematopoietic immune cells contribute to this protective effect, we generated bone marrow chimeras by reconstituting irradiated WT recipients with either WT or CYLD KI bone marrow. In this setting, EAE severity was comparable between groups, indicating that altered hematopoietic cell function alone does not account for the reduced disease susceptibility in CYLD KI mice.

Interestingly, MALT1-mediated CYLD cleavage has been implicated in regulating endothelial barrier integrity (Klei et al, 2016). Specifically, thrombin-induced CYLD cleavage disrupts its role in maintaining microtubule stability in endothelial cells. It is therefore plausible that differences in blood–brain barrier (BBB) permeability contribute to the EAE phenotype in CYLD KI mice. However, chimeric mice in which only the non-hematopoietic compartment expressed cleavage-resistant CYLD also failed to show altered disease severity. The fact that none of the bone marrow chimera experimental settings still showed differences in EAE disease development between WT and CYLD KI mice suggests that full protection in CYLD KI mice requires both hematopoietic and non-hematopoietic compartments. Nonetheless, we cannot exclude the possibility that irradiation-induced BBB disruption and glial activation in recipient mice may have masked potential differences in BBB integrity and EAE susceptibility between control and CYLD KI chimeras (Gourdain et al, 2012)

Surprisingly, the reduced EAE sensitivity of CYLD KI mice was lost upon co-housing with WT mice in the same cage, suggesting a role for the intestinal microbiota. Indeed, separately housed CYLD KI mice displayed a distinct gut microbial composition compared to controls, with a trend toward reduced Firmicutes and increased Bacteroidetes. Alterations in the F/B ratio are commonly associated with gut dysbiosis and various health conditions. In the context of multiple sclerosis and EAE, both increased and decreased F/B ratios have been linked to disease progression (Regen et al, 2021; Kumar et al, 2016; Berer et al, 2011, 2017). These observations support the hypothesis that the reduced neuroinflammation in CYLD KI mice may be driven by microbiota-dependent mechanisms.

Because host–microbiota interactions are strongly influenced by intestinal barrier integrity, we next assessed whether expression of cleavage-resistant CYLD affects epithelial homeostasis. Histological analysis of the small intestine and colon revealed no detectable abnormalities in intestinal tissue architecture and goblet cell distribution. Moreover, expression of the Paneth cell antimicrobial peptide lysozyme and intestinal permeability were comparable between WT and CYLD KI mice under steady-state conditions. These findings indicate that the altered microbial composition observed in CYLD KI mice is unlikely to result from defects in intestinal barrier structure or permeability. In this context, it is worth noting that acute irradiation used in bone marrow chimera experiments may also alter microbiome composition (Jameus et al, 2024), potentially influencing the outcome and interpretation of these studies. The observed link between MALT1-mediated CYLD cleavage, gut microbiota composition, and disease susceptibility is reminiscent of the role of CARD9-mediated MALT1 signaling in shaping the gut microbiome, including its bacterial and fungal communities and metabolic output (Luo et al, 2020). Together, these findings suggest the existence of a CBM-CYLD signaling axis that influences gut microbial ecology. Notably, multiple sclerosis patients with high disease activity and elevated intestinal Th17 cell levels have been reported to exhibit a higher F/B ratio compared to healthy individuals (Cosorich et al, 2022). Moreover, healthy individuals harbor T cells with high affinity for central nervous system autoantigens, which can become pathogenic following microbiota shifts (Berer et al, 2017). Based on these insights, we hypothesized that the unique microbiota of separately housed CYLD KI mice might reduce EAE susceptibility by modulating intestinal Th1 and Th17 cell populations. However, no significant differences in intestinal CD4⁺ T cell subsets were observed among separately housed CYLD KI, co-housed CYLD KI, and control mice prior to EAE induction.

Taken together, our findings demonstrate that MALT1-mediated CYLD cleavage is dispensable for lymphocyte development, activation, and effector function under both homeostatic and inflammatory conditions. While CYLD cleavage does not appear to influence canonical immune signaling pathways, it may contribute to disease susceptibility through mechanisms involving tissue-specific or environmental factors, such as the gut microbiota or blood–brain barrier integrity. Importantly, our study provides in vivo evidence that selective inhibition of a single MALT1 substrate can uncouple pathogenic outcomes from essential immune functions. This highlights the potential of substrate-specific targeting as a refined therapeutic strategy, offering a conceptual alternative to broad MALT1 inhibition, which is often associated with systemic immune dysregulation. Further studies using refined in vivo models will be essential to fully elucidate the context-dependent roles of CYLD cleavage in immune regulation and neuroinflammation.

## Methods

**Table:** Reagents and tools table

Reagent/resource	Reference or source	Identifier or catalog number
**Experimental models**		
C57BL/6 CD45.2 CYLD R321A Knock-In mice	Cyagen Biosciences (This study)	NA
C57BL/6 CD45.2 CYLD WT mice	Cyagen Biosciences (This study)	NA
B6.SJL-PtprcaPepcb/BoyJ CD45.1 mice	IRC Transgenic core facility	NA
**Antibodies**		
Anti-mouse CD16/CD32 (2.4G2)	BD Biosciences	Cat# 553142
eFluor450 anti-mouse CD3 (17A2)	eBioscience	Cat# 48-0032-82
PE-Cy5 anti-mouse CD3 (145-2C11)	Cytek Biosciences	Cat# SKU 55-0031-U025
FITC anti-mouse CD4 (GK1.5)	BD Biosciences	Cat# 553047
FITC anti-mouse CD4 (GK1.5)	eBioscience	Cat# 11-0041-82
BUV395 anti-mouse CD4 (GK1.5)	BD Biosciences	Cat# 563790
PE-Cy7 anti-mouse CD8a (53-6.7)	eBioscience	Cat# 25-0081-82
PerCP-Cy5.5 anti-mouse CD25 (PC61)	eBioscience	Cat# 45-0251-82
BUV395 anti-mouse CD25 (PC61)	BD Biosciences	Cat# 564022
APC-eFluor780 anti-mouse CD44 (IM7)	eBioscience	Cat# 47-0441-82
Alexa Fluor 700 anti-mouse CD44 (IM7)	BD Biosciences	Cat# 560567
APC anti-mouse CD62L (MEL-14)	eBioscience	Cat# 17-0621-81
PE anti-mouse CD62L (MEL-14)	eBioscience	Cat# 12-0621-82
PE anti-mouse Foxp3 (FJK-16s)	eBioscience	Cat# 12-5773-82
APC anti-mouse Foxp3 (FJK-16s)	eBioscience	Cat# 17-5773-82
FITC anti-mouse CD45R/B220 (RA3-6B2)	BD Biosciences	Cat# 553087
eFluor450 anti-mouse CD21/CD35 (4E3)	eBioscience	Cat# 48-0212-82
PE-Cy7 anti-mouse CD23 (B3B4)	eBioscience	Cat# 25-0232-81
PE-Cy7 anti-mouse IFNγ (XMG1.2)	eBioscience	Cat# 25-7311-82
PerCP-Cy5.5 anti-mouse IL-17 (TC11-18H10)	eBioscience	Cat# 45-7177-80
BV605 anti-mouse IL-17 (TC11-18H10)	BD Biosciences	Cat# 564169
APC anti-mouse IL-4 (11B11)	eBioscience	Cat# 17-7041-81
Anti-mouse CD120b BV421 (TR75-89)	BD	Cat# 564088
Anti-mouse CD152 PE-eFluor 610 (UC10-4B9)	eBioscience	Cat# 61-1522-82
HOIL-1 rabbit polyclonal	Merck	Cat# HPA024185
CYLD (clone E10)	Santa Cruz Biotechnology	Cat# sc-74435
Regnase-1 (clone 604421)	R&D Systems	Cat# MAB7875
IκBα (clone C21)	Santa Cruz Biotechnology	Cat# sc-371-G
Phospho-IκBα (clone 5A5)	Cell Signaling Technology	Cat# 9246 L
JNK (clone FL)	Santa Cruz Biotechnology	Cat# 9252
Phospho-JNK	Invitrogen	Cat# 44-682 G
ERK	Cell Signaling Technology	Cat# 9102
Phospho-ERK	Cell Signaling Technology	Cat# 9101
p38	Cell Signaling Technology	Cat# 9212
Phospho-p38 (3D7)	Cell Signaling Technology	Cat# 9215
Actin-HRP (C4)	Santa Cruz Biotechnology	Cat# sc-47778
HOIL-1 rabbit polyclonal	Merck	Cat# HPA024185
CYLD (clone E10)	Santa Cruz Biotechnology	Cat# sc-74435
Regnase-1 (clone 604421)	R&D Systems	Cat# MAB7875
IκBα (clone C21)	Santa Cruz Biotechnology	Cat# sc-371-G
Phospho-IκBα (clone 5A5)	Cell Signaling Technology	Cat# 9246 L
JNK (clone FL)	Santa Cruz Biotechnology	Cat# 9252
Phospho-JNK	Invitrogen	Cat# 44-682 G
ERK	Cell Signaling Technology	Cat# 9102
Phospho-ERK	Cell Signaling Technology	Cat# 9101
p38	Cell Signaling Technology	Cat# 9212
Phospho-p38 (3D7)	Cell Signaling Technology	Cat# 9215
BD Pharmingen™ Purified NA/LE Hamster Anti-Mouse CD3e	BD Biosciences	Cat# 553057
BD Pharmingen™ Purified NA/LE Hamster Anti-Mouse CD28	BD Biosciences	Cat# 553294
Anti-TNP Purified Mouse IgM, k Isotype Control Clone G155-228	BD Pharmingen™	Cat# 555581
Anti-TNP Purified Mouse Mouse IgG1 λ Isotype Control	BD Pharmingen™	Cat# 553485
Goat anti-mouse IgM-HRP	Thermo Fisher Scientific	Cat# 62-6820
Goat anti-mouse IgG1-HRP	Southern Biotechnology	Cat# 1071-05
**Oligonucleotides and other sequence-based reagents**		
mCyld-gRNA-F primer	Integrated DNA Technologies	GCTCTTGTCATTGATTGCTAAACTATTTCTC
mCyld-gRNA-R primer	Integrated DNA Technologies	CCTGACAATTCATGGGGTGGC
16S rRNA V3-V4 primer 341 F	LGC Genomics	CCTACGGGNGGCWGCAG
16S rRNA V3-V4 primer 785Rmod	LGC Genomics	GACTACHVGGGTATCTAAKCC
Ubc forward primer	Integrated DNA Technologies	aggtcaaacaggaagacagacgta
Ubc reverse primer	Integrated DNA Technologies	tcacacccaagaacaagcaca
Hprt1 forward primer	Integrated DNA Technologies	AGTGTTGGATACAGGCCAGAC
Hprt1 reverse primer	Integrated DNA Technologies	cgtgattcaaatccctgaagt
GAPDH forward primer	Integrated DNA Technologies	TGAAGCAGGCATCTGAGGG
GAPDH reverse primer	Integrated DNA Technologies	CGAAGGTGGAAGAGTGGGAG
RPL13a forward primer	Integrated DNA Technologies	CCTGCTGCTCTCAAGGTT
RPL13a reverse primer	Integrated DNA Technologies	TGGTTGTCACTGCCTGGTACTT
Lyz1 forward primer	Integrated DNA Technologies	gccaaggtctaacaatcgttgtgagttg
Lyz1 reverse primer	Integrated DNA Technologies	cagtcagccagcttgacaccacg
**Chemicals, Enzymes and other reagents**		
2-Mercaptoethanol (50 mM)	Gibco	Cat# 31350010
4 kDa FITC-dextran	Tdb labs (sweden)	Cat. #FCMD4
RPMI 1640 w/o NaHCO3, w/ L-glutamine	Gibco	Cat. # 52400025
ACK lysis buffer	Gibco	Cat. # A1049201
Collagenase VIII	Sigma	Cat. #C2139
HBSS	Gibco	Cat. #14185-045
EDTA	Vel	Cat. #9249
1× D-PBS liquid w/o Ca and Mg	Gibco	Cat. #14190-094
Brefeldin A	Sigma-Aldrich	Cat# B7651
MyTaq DNA polymerase	Bioline	Cat# BIO-21105
Fetal bovine serum	Bodinco	NA
Ionomycin calcium salt (from Streptomyces conglobatus)	Sigma-Aldrich	Cat# 407952
L-Glutamine (200 mM)	Lonza	Cat# BE17-605F
Penicillin-streptomycin solution stabilized	Sigma-Aldrich	Cat# P4333
Phorbol 12-myristate 13-acetate (PMA)	Sigma-Aldrich	Cat# P8139
Sodium pyruvate solution (100 mM)	Sigma-Aldrich	Cat# S8636
Trypan blue	Sigma-Aldrich	Cat# 1.11732
BD Horizon Brilliant Stain Buffer	BD	Cat# 566349
Fixable Viability Dye eFluor506	eBioscience	Cat# 65-0866-14
Fixable Viability Dye eFluor780	eBioscience	Cat# 65-0865-18
Cell proliferation dye eFluor450	eBioscience	Cat# 65-0842-90
MOG35–55 peptide	Sigma Genosys	Custom peptide (MEVGWYRSPFSRVVHLYRNGK)
Complete Freund’s Adjuvant	Sigma-Aldrich	Cat# F-5881
Mycobacterium tuberculosis H37RA	Difco	Cat# 231141
Pertussis Toxin from Bordetella Pertussis	Sigma-Aldrich	Cat# P-2980
TNP-AECM-Ficoll	Biosearch Technologies	Cat# F-1300F
TNP-KLH	Biosearch Technologies	Cat# T-5060
Aluminum hydroxide	Thermo Fisher Scientific	Cat# 77161
TMB substrate	eBioscience	Cat# 00-4201-56
H_2_SO_4_ (1 m)	In house	NA
TNP-BSA (Bovine Serum Albumin)	Biosearch Technologies	Cat# T-5050
Entellan mounting medium	Merck Millipore	Cat# MERC1.07961.0100
Alcian Blue 8GX	Thermo Fisher Scientific	Cat# J60122.14
Hematoxylin and Eosin	Proteintech	Cat# PK10031
**Software**		
Axio Scan.Z1 slide scanner	Zeiss	
GraphPad Prism	GraphPad Software v8-v10	
Zen Lite	Zeiss Microscopy.	
qBase+	Biogazelle	
flowjo_v10.7.2 Software or recent	FlowJo LLC, a BD Company (Tree Star Inc)	
QIIME2	QIIME2 development team	
Emperor	QIIME2 visualization tool	
DADA2	Bioconductor	
RStudio	RStudio	
QuPath	University of Edinburgh	
BD FACSDiva	BD Biosciences	
Genstat	VNS International	
**Other**		
Fixation/Permeabilization Solution Kit	BD	Cat# 554714
FoxP3/Transcription Factor Staining Buffer Set	eBioscience	Cat# 00-5523-00
UltraComp eBeads Compensation Beads	Invitrogen	Cat# 01-2222-42
Cell counting chamber without clamps, dark lined, Bürker	MARIENFELD	Cat# 0640210
Tissue culture plate, 48 well, flat bottom with low evaporation lid	Falcon	Cat# 353078
IL-2 Mouse Uncoated ELISA kit	eBioscience	Cat# 88-7120-88
Bio-Plex Pro Mouse Cytokine IL-17A set, 1 ×96-well	Bio-Plex	Cat# 171-G5013M
Bio-Plex Pro Mouse Cytokine IFN-gamma set, 1 ×96-well	Bio-Plex	Cat# 171-G5017M
Bio-Plex Pro Mouse IL-10 set, 1x96well	Bio-Plex	Cat# 171-G5009M
RNeasy mini kit	Qiagen	Cat# 74104
SensiFAST cDNA synthesis kit	GC Biotech	Cat# BIO-650504
SensiFAST SYBR No-ROX kit	GC Biotech	Cat# CSA-01190
MagniSort Mouse CD4 T cell Enrichment Kit	Invitrogen	Cat# 8804-6821-74
MagniSort Mouse B cell Enrichment Kit	Invitrogen	Cat# 8804-6827-74
Bio-Plex 200 System	Bio-Rad	Bio-Plex 200 System
FLUOstar Omega Plate Reader	BMG LABTECH	FLUOstar Omega Plate Reader
LightCycler 480	Roche	LightCycler 480
FastPrep bead-beating system	MP Biomedicals	FastPrep bead-beating system
PowerLyzer homogenizer	Qiagen	PowerLyzer homogenizer
LSRII Flow Cytometer	BD Biosciences	
LSR Fortessa Flow Cytometer	BD Biosciences	
Illumina MiSeq	Illumina	
Amersham Imager 600	Cytiva	

### Ethics statement

All mouse experiments were approved by the Ethics Committee for Laboratory Animal Welfare of the Faculty of Sciences at Ghent University (approval numbers: LN0144, EC2020-070, EC2020-071, EC2022-042, and EC2025-158).

### Mice

C57BL/6 CYLD KI mice, in which arginine at position 321 of CYLD was replaced by alanine (R321A), were generated by Cyagen Biosciences Inc. (Santa Clara, CA, USA) using CRISPR/Cas9-mediated genome editing in fertilized eggs. The oligonucleotide donor contained the R321A mutation in exon 4 and a silent NaeI restriction site (AGAGGT to GCCGGC), flanked by 120 bp homology arms: TTTGCTGTTTCAGATAGCGTGACACAGGAAAGGAGGCCTCCCAAACTTGCCTTTATGTCAGCCGGCGTAGGTGACAAAGGTTCATCTAGTCATAATAAACCAAAGGTTACAGGTATGGAGCTATTC. Cas9 mRNA, guide RNA (GCCTTTATGTCAAGAGGTGTAGG; generated by in vitro transcription), and the oligo donor were co-injected into fertilized eggs. Pups were genotyped by PCR using the following primers: mCyld-gRNA-F: GCTCTTGTCATTGATTGCTAAACTATTTCTC; mCyld-gRNA-R: CCTGACAATTCATGGGGTGGC). PCR products were analyzed by sequencing and NaeI restriction digestion, yielding the following fragment sizes: WT allele 360 bp; mutant allele 129 bp and 231 bp.

Male B6.SJL-PtprcaPepcb/BoyJ CD45.1 WT mice were used to generate bone marrow chimeras. Age- and sex-matched mice (8–16 weeks old) were used for all in vitro experiments. For EAE and gut microbiome studies, age-matched male non-littermate WT and CYLD KI mice were either co-housed in the same cage from weaning or housed in separate cages. Experimental WT and CYLD KI mice were derived from multiple homozygous F2 breeding pairs, which originated from F1 heterozygous (HET) x HET, HET x CYLD KI, or HET x WT crosses to minimize genetic drift. EAE was induced at least 4 weeks after the start of co-housing. All mice were housed under specific pathogen-free conditions in accordance with institutional guidelines.

In all experiments described in this manuscript, mice were randomly assigned to treatment groups. A minimum of three mice per group was analyzed, depending on the availability of the appropriate genotypes. Investigators were not blinded to group allocation during data analysis. No data points were excluded, during statistical analysis.

### Generation of bone marrow chimeras

To generate hematopoietic chimeras, bone marrow cells were isolated from CD45.1 WT donor mice. CD45.2 WT control and CD45.2 CYLD KI recipient mice were lethally irradiated with two doses of 4.5 Gy (4 h apart). At least 12 h after the final irradiation, recipients received an intravenous injection of 2 × 10⁶ CD45.1 donor bone marrow cells. Similarly, reciprocal chimeras were generated by reconstituting lethally irradiated CD45.1 WT recipients with bone marrow from CD45.2 WT or CD45.2 CYLD KI donors. Chimeric mice were monitored every 5 days. Six weeks post-transplantation, successful engraftment was confirmed by assessing flow cytometric analysis of circulating CD45.1^+^ lymphocytes in peripheral blood, indicating effective hematopoietic reconstitution.

### Flow cytometry

Single-cell suspensions from the thymus, spleen, and cervical lymph nodes of 8–14 weeks old mice were prepared as previously described (Skordos et al, 2021) For flow cytometry analysis of T cells and Tregs in naïve CYLD KI mice, cells were stained with Fixable Viability Dye eFluor506 (eBioscience, Thermo Fisher Scientific, Waltham, MA, USA), anti-CD16/CD32 Fc block (clone 2.4G2; BD Biosciences), and the following antibodies: anti-CD3-eFluor450 (clone 17A2), anti-CD4-FITC (clone GK1.5), anti-CD8a-PE-Cy7 (clone 53-6.7), anti-CD25-PercP-cy5.5 (clone PC61), anti-CD44-APC-eFluor780 (clone IM7), and anti-CD62L-APC (clone MEL-14) (all from eBioscience unless otherwise stated). Staining was performed for 20 min on ice. Cells were then permeabilized using the Foxp3 buffer set (eBioscience) according to the manufacturer’s instructions, followed by intracellular staining with anti-Foxp3-PE (clone FJK-16s) for 30 min. All steps were conducted on ice. CTLA-4 and TNFR2 expression on CYLD KI Tregs were assessed following the protocol described by Demeyer et. al. (Demeyer et al, 2020).

For analysis of marginal zone and follicular B cells, splenocytes were stained with Fixable Viability Dye eFluor506, anti-CD16/CD32 Fc block, and the following antibodies: anti-CD3-PE-Cy5 (clone 145-2C11; Cytek Biosciences), anti-CD45R/B220-FITC (clone RA3-6B2; BD Biosciences), anti-CD21/CD35-eFluor450 (clone 4E3; eBioscience), and anti-CD23-PE-Cy7 (clone B3B4; eBioscience) for 20 min on ice. For intracellular cytokine staining (ICS), splenocytes and cervical lymph node cells were cultured in complete RPMI 1640 medium (supplemented with 10% FCS, sodium pyruvate, L-glutamine, antibiotics, and β-mercaptoethanol) and stimulated with PMA (50 ng/mL), ionomycin (500 ng/mL), and brefeldin A (10 µg/mL) for 4 h at 37 °C. Cells were stained with Fixable Viability Dye eFluor506, anti-CD16/CD32 Fc block, anti-CD3-eFluor450, anti-CD4-FITC, anti-CD8a-PercP-cy5.5 (clone 53-6.7; eBioscience), anti-CD44-APC eFluor780, for 20 min. After fixation and permeabilization for 30 min using the BD Cytofix/Cytoperm Fixation/Permeabilization Solution Kit (BD Biosciences, San Diego, CA, USA), intracellular cytokines were stained with anti-IFNγ-PE-Cy7 (clone XMG1.2; eBioscience), anti-IL-17-BV605 (clone TC11-18H10; BD Biosciences) and anti-IL-4-APC (clone 11B11; eBioscience) for 30 min.

To assess bone marrow engraftment efficiency in CD45.1-reconstituted CD45.2 control WT and CYLD KI mice, blood samples were collected from the tail vein. Lymphocytes were stained with Fixable Viability Dye eFluor506, anti-CD16/CD32 Fc block, anti-CD3-PE-Cy5, anti-CD45R/B220-FITC, anti-CD45.1-BV605 (clone A20; BioLegend), and anti-CD45.2-BUV737 (clone 104; BD Biosciences) for 20 min on ice following a protocol similar to Skordos et al. (Skordos et al, 2021).

Flow cytometry data were collected using an LSRII or LSR Fortessa system (BD Biosciences) equipped with five lasers. Data analysis were performed using FlowJo software (versions 10.7.2-10.10.0; Tree Star, Inc., Ashland, OR, USA).

### Analysis of proliferation and cytokine production by activated CD4^+^ T Cells

CD4^+^ T cells were isolated from control WT and CYLD KI mice using the MagniSort Mouse CD4 T cell Enrichment Kit (Invitrogen™; Cat. #65-0842-90). Following isolation, cells were labeled with 10 μM Cell Proliferation Dye eFluor450 (eBioscience; Cat. #8804-6821-74) according to the manufacturer’s instructions. Labeled cells were cultured for 72 h in RPMI 1640 medium supplemented with 5 µg/mL plate-bound anti-CD3ε (clone 145-2C11; BD Pharmingen) and 5 µg/mL soluble anti-CD28 (clone 37.51; BD Pharmingen). After 72 h, T-cell activation and proliferation were assessed by flow cytometry after surface staining of cells with Fixable Viability Dye eFluor780 (eBioscience), anti-CD4-FITC, anti-CD25-PerCP-Cy5.5, anti-CD62L-PE, anti-CD69-APC, and anti-CD44-Alexa Fluor 700, along with anti-CD16/CD32 Fc block, as described in the flow cytometry section.

For cytokine production assays, 5 × 10⁵ CD4^+^ T cells were seeded into 48-well plates and cultured under the same conditions (anti-CD3ε and anti-CD28). After 20–24 h, culture supernatants were collected and stored at −20 °C. IL-2 levels were quantified using a mouse IL-2 ELISA kit (eBioscience; Cat. # 88-7024-88), following the manufacturer’s protocol.

### Study of small intestinal lamina propria T cells

To isolate immune cells from the small intestinal lamina propria of naïve mice, intestines were excised, flushed with cold 2% FCS in HBSS to remove fecal content, and cleared of Peyer’s patches and fat. The cleaned tissue was turned inside out and incubated twice in a dissociation buffer (HBSS without calcium and magnesium, 2 mM EDTA, 5% FBS) at 37 °C for 20 min per cycle in a water bath with continuous rotation at 100 rpm to remove epithelial cells. Following epithelial removal, tissues were washed with warm HBSS, cut into small pieces, and transferred to 50 mL tubes for enzymatic digestion. Fragments were incubated in RPMI 1640 containing 2% FCS and 0.6 mg/mL collagenase VIII at 37 °C for up to 10 min with gentle rotation. Digestion was stopped by adding PBS supplemented with 10% FCS and 2 mM EDTA. The resulting cell suspension was filtered through a 70 µm strainer to obtain a single-cell suspension, centrifuged at 400×*g* for 4 min at 4 °C, and resuspended in T cell medium for downstream analysis.

Tregs were identified by flow cytometry using anti-FoxP3-APC (clone FJK-16s; eBioscience), as described in the flow cytometry section. Additional surface staining of single-cell suspensions included Fixable Viability Dye eFluor506, anti-CD16/CD32, anti-CD44-AF700, anti-CD4-APC-eFluor780, anti-CD62L-PE, anti-CD3-PE-Cy5, anti-CD8-PE-Cy7, anti-CD25-BUV395, and anti-CD45-FITC (clone 30-F11; Thermo Fisher Scientific). To assess cytokine production, single-cell suspensions were stimulated with P/I, and brefeldin A for 4 h. After stimulation, cells were stained with Fixable Viability Dye eFluor506, anti-CD16/CD32, anti-CD3-PE-Cy5, anti-CD4-BUV395, anti-CD8a-BV605, anti-CD25-BUV395, anti-CD44-PE, and anti-CD45-FITC. Intracellular cytokine staining was then performed using anti-IFN-γ-PE-Cy7 and anti-IL-17 PerCP-Cy5.5, as described in the flow cytometry section.

### Induction and assessment of hapten-specific humoral immune responses

To induce T cell-independent type 2 (TI-2) and T cell-dependent (T-D) humoral immune responses in control WT and CYLD KI mice, we followed the protocol described by Skordos et al (Skordos et al, 2023). For TI-2 responses, TNP-AECM-Ficoll (Biosearch Technologies; Cat. #F-1300-10) was dissolved in PBS at a concentration of 250 µg/mL. Mice were injected intraperitoneally (i.p.) with 25 µg of TNP-AECM-Ficoll. For T-D responses, TNP-KLH (Biosearch Technologies; Cat. # T-5060-25) was adsorbed onto aluminum hydroxide (Thermo Fisher Scientific; Cat. #77161) in a 1/1 (v/v) ratio, yielding a final concentration of 500 µg/mL. Mice were then injected i.p. with 100 µg TNP-KLH and monitored for 28 days.

### Evaluation of serum antibody levels

Serum anti-TNP antibody levels were measured following the protocol described by Skordos et al. (Skordos et al, 2023). Blood samples were collected at the indicated time points following T cell-dependent or T cell-independent immunization. Samples were allowed to clot at room temperature, and serum was isolated and stored at −20 °C until analysis. TNP-specific IgM and IgG1 levels were measured using Nunc MaxiSorp 96-well flat-bottom plates coated overnight at 4 °C with 10 µg/mL TNP-BSA (Biosearch Technologies). Nonspecific binding was blocked by incubating plates with 0.5% BSA in PBS for 1.5 h at room temperature. Serum samples were diluted starting at 1:10,000 in 0.05% Tween-20 in PBS and incubated for 2 h at 37 °C. Standard curves were generated using serial dilutions of purified anti-TNP mouse IgM (κ isotype control; BD Pharmingen, clone G155-228) and anti-TNP mouse IgG1 (λ isotype control; BD Pharmingen, clone A111-3) in the same buffer. For detection, IgM-HRP (Thermo Fisher Scientific) was added at a dilution of 1:2000 and incubated for 2 h at room temperature. IgG1-HRP (Southern Biotechnology) was used at a 1:4000 dilution and incubated for 1 h at 37 °C. TMB substrate (eBioscience) was then added, and the enzymatic reaction was stopped with 1 M H_2_SO_4_. Optical density was measured at 450 nm using a microplate reader.

### Assessment of intestinal permeability

Naïve WT and CYLD KI mice were fasted for 4 h prior to the experiment. Mice were then orally gavaged with 4-kDa FITC-dextran (5 mg/g body weight in PBS, FD4, Sigma-Aldrich). Blood samples were collected via mandibular bleeding 2 and 4 h after gavage, incubated for 30 min at RT, and serum was separated by centrifugation at 3000 rpm for 30 min at 4 °C. Fluorescence was measured in 96-well plates at excitation wavelength 485 nm and emission wavelength 520 nm using FLUOstar Omega (BMG LABTECH). Concentrations were calculated using a standard range between 125 and 8000 ng/ml. Mouse serum without 4 kDa FITC-dextran administration was used to subtract the background.

### EAE induction and assessment

EAE was induced using the MOG_35–55_ peptide (MEVGWYRSPFSRVVHLYRNGK), synthesized by Sigma Genosys. Non-littermate WT and CYLD KI male mice, either co-housed or separately caged, were sedated and subcutaneously immunized at the flanks near the hindlimbs with an emulsion containing 200 μg of MOG₃₅–₅₅ peptide in 200 μL of sterile PBS mixed 1:1 with Complete Freund’s Adjuvant (CFA; Sigma-Aldrich), supplemented with 10 mg/mL *Mycobacterium tuberculosis* H37RA (BD Biosciences). To enhance immunogenicity, 50 ng of pertussis toxin (Sigma-Aldrich) in 200 μL of sterile PBS was administered i.p. at the time of immunization and again 48 h later. Mice were monitored daily for 25 days for changes in body weight and clinical signs of disease, as previously described^12^. Clinical scores were assigned in a blinded manner using a 0-6 scale with half-point increments for intermediate phenotypes: 0, normal; 1, tail weakness; 2, complete tail tonicity loss; 3, partial hind limb paralysis; 4, complete hind limb paralysis; 5, forelimb paralysis or moribund state; 6, death.

Separately caged, lethally irradiated, non-littermate male CD45.2 WT and CD45.2 CYLD KI, descended from heterozygous or heterozygous-bred grandparents, were reconstituted with CD45.1 WT bone marrow cells, as previously described. Conversely, lethally irradiated CD45.1 WT mice were reconstituted with bone marrow from CD45.2 WT or CD45.2 CYLD KI mice. Ten weeks post-reconstitution, mice were actively immunized with MOG_35–55_ peptide in CFA using the same protocol as above-mentioned and monitored for EAE development over 25 days.

### T cell recall assay and analysis of MOG-autoreactive T cells

T-cell recall responses were assessed in splenocytes and draining lymph node cells 10 days post-EAE induction. Following isolation, erythrocytes were lysed using ACK buffer to minimize background interference. Cells were plated in flat-bottom 96-well plates at a density of 4 × 10⁵ cells/well in DMEM supplemented with 5% FCS, L-glutamine, non-essential amino acids, β-mercaptoethanol, and Pen/Strep. Cultures were stimulated with 30 µg/mL MOG_35–55_ peptide and incubated for 48 h (splenocytes) or 96 h lymph node cells). Supernatants were collected for quantification of IFN-γ, IL-17, and IL-10 using the Bio-Plex Pro cytokine assay kit on the Bio-Plex^TM^ 200 system. To further characterize MOG-specific Th1 and Th17 responses, splenocytes and draining lymph node cells from day 10 post-immunization were cultured in RPMI 1640 (as described in the passive EAE protocol) with 30 µg/mL MOG_35–55_ for 72 h. Brefeldin A (BFA) (10 µg/mL) was added during the final 4 h to facilitate intracellular cytokine accumulation. Cells were then stained using an intracellular cytokine panel and analysed by flow cytometry to measure IFN-γ and IL-17 production in CD4^+^ T cells. Additionally, Treg frequencies in spleen and draining lymph nodes were evaluated by flow cytometry at day 10 post-EAE induction.

### Extraction of bacterial DNA

Fresh fecal samples were collected, immediately snap-frozen in liquid nitrogen, and stored at −20 °C until further processing. Total DNA was extracted from the pellet of a 1 mL fecal slurry using bead beating with a PowerLyzer (Qiagen, Venlo, NL, EU) and phenol/chloroform extraction, as previously described (Miclotte et al, 2020). Briefly, 1 mL of Tris/HCl lysis buffer (100 mM, pH 8) and 200 mg of glass beads (Sartorius, Göttingen, DE, EU) were added to the sample. Cells were mechanically lysed by multidirectional bead beating for two times 40 s at 1600 rpm using a FastPrep system (MP Biomedicals, Santa Ana, CA, USA). Glass beads were removed by centrifugation for 5 min at maximum speed. DNA was purified via phenol-chloroform extraction and precipitated by adding one volume of ice-cold isopropanol and 1:10 volume of 3 M sodium acetate. Samples were incubated at −20 °C for at least 1 h, followed by centrifugation for 30 min at maximum speed. The supernatant was discarded, and the DNA pellet was air-dried and resuspended in 100 µL TE buffer. DNA quality was verified by electrophoresis on a 1.5% (w/v) agarose gel.

### 16S rRNA gene amplification and sequencing

Genomic DNA (10 μL) was sent to LGC Genomics GmbH (Berlin, Germany) for library preparation and amplicon sequencing of the V3-V4 region of the 16S rRNA gene. Sequencing was performed on an Illumina MiSeq platform using v3 chemistry with primers 341F (5′-CCTACGGGNGGCWGCAG-3′) and 785Rmod (5′-GACTACHVGGGTATCTAAKCC-3′) (Klindworth et al, 2013). A mock microbial community was included in triplicate to assess sequencing quality. Raw Illumina reads were processed using the DADA2 pipeline. Primer sequences were removed, reads were trimmed and dereplicated, and forward and reverse reads were merged. An Amplicon Sequence Variant table was generated, chimeric sequences were filtered out, and the read counts were checked through the pipeline. Taxonomic classification was performed using the Silva database (version 132) (Glöckner, 2019).

### Microbial community composition analysis

All sequences were quality-filtered to remove reads with a quality score of <20. Samples were rarefied to 10,000 sequences per sample. Alpha diversity was calculated using the Shannon diversity index in QIIME2 (Bolyen et al, 2019). Beta diversity was calculated using Bray–Curtis distances, and principal component analysis was performed using QIIME2 and visualized with Emperor (Vázquez-Baeza et al, 2013). Pairwise differences in alpha diversity were tested using the nonparametric Mann–Whitney *U*-test. Differences in beta diversity were evaluated using the Adonis test based on Bray–Curtis distance metrics. Statistical analysis on the microbiome dataset was performed in RStudio, with significance defined at α = 0.05. Trends with *p*-values slightly above this threshold were also considered potentially informative (Wasserstein et al, 2019).

### Western blotting

Single-cell suspensions from spleens were prepared by mechanical dissociation and filtration through a 70 µm strainer. CD4^+^ T cells and B cells were purified from pooled spleens using the MagniSort Mouse CD4 T and B cell Enrichment Kits (Invitrogen™; Cat. #65-0842-90 and #8804-6827-74, respectively). Splenocytes or purified CD4^+^ T and B cells were stimulated for the indicated times with 200 ng/mL PMA and 1 mM Ionomycin. Cells were collected by centrifugation at 4 °C, washed with PBS, and lysed in E1A buffer (50 mM Hepes pH 7.6, 250 mM NaCl, 5 mM EDTA, 0.5% (vol/vol) NP-40) supplemented with phosphatase inhibitors (NaF, Na3VO4) and protease inhibitors (aprotinin, leupeptin, Pefabloc SC). Lysates were incubated on ice for 15 min and cleared by centrifugation at 13,000×*g* for 10 min. Protein concentrations were determined using the Bradford assay. Lysates were heated for 10 min at 95 °C, separated by SDS-PAGE, and transferred via semi-dry blotting. Proteins were detected using enhanced chemiluminescence (Perkin-Elmer Life Sciences). The following antibodes were used: anti-HOIL-1 (polyclonal; Merck; Cat. # HPA024185), anti-CYLD (clone E10; Santa Cruz Biotechnology Inc.), anti-Regnase-1 (clone 604421; R&D systems), anti-IκBα (clone C21; Santa Cruz Biotechnology Inc.), anti-P-IκBα (clone 5A5; Cell Signaling Technology), anti-JNK (clone FL; Santa Cruz Biotechnology Inc.), anti-P-JNK1/JNK2 (polyclonal; Invitrogen; Cat. # 44-682 G), anti-ERK (polyclonal; Cell Signaling Technology; Cat. # 9102), anti-P-ERK (polyclonal; Cell Signaling Technology; Cat. # 9101), anti-p38 (polyclonal; Cell Signaling Technology; Cat. # 9212), anti-P-p38 (clone 3D7; Cell Signaling Technology; Cat # 9215S), and anti-Actin-HRP (clone C4; Santa Cruz Biotechnology Inc.; Cat # sc-47778).

### Histological analysis of intestinal tissues

Segments of the small intestine and colon were harvested from naïve WT and CYLD KI mice, flushed with cold PBS, and fixed in 4% paraformaldehyde overnight at 4 °C. Tissues were subsequently dehydrated, embedded in paraffin, and sectioned at 5 µm thickness. For morphological assessment, sections were stained with hematoxylin and eosin (H&E) according to standard protocols. To visualize mucus-producing goblet cells, adjacent sections were stained with Alcian Blue (pH 2.5). Slides were mounted with Entellan (MERC1.07961.0100, Merck Millipore), imaged with the Axio Scan.Z1 (Zeiss), and analyzed with Zen Lite (Zeiss) and QuPath software. Representative images were acquired at 200 and 50 µm scales.

### Quantification of Paneth cell lysozyme expression

Small intestinal ileum tissue samples (0.015 g) were collected from naïve-state WT and CYLD KI mice, living separately, and immediately snap-frozen in liquid nitrogen. Total RNA was extracted using RNeasy Plus kits (Qiagen) according to the manufacturer’s instructions. DNA was synthesized using a SensiFast cDNA synthesis Kit (BIO-650504, GC Biotech) according to the manufacturer’s instructions. Quantitative PCR was performed using LightCycler 480 (Roche) with SensiFast SYBR No-Rox kit (CSA-01190, GC Biotech) using a total of 10 ng cDNA and 1 µM of specific primer in a total volume of 10 µl. Samples were analyzed in triplicate. Analysis was done using qBase^+^ software (Biogazelle, Gent, Belgium). Values were normalized to the appropriate amount of reference genes, as determined by geNorm analysis in the qBase software. The following primers were used: *Ubc* forward, *aggtcaaacaggaagacagacgta*; Ubc reverse, *tcacacccaagaacaagcaca*, Hprt1 forward, *AGTGTTGGATACAGGCCAGAC*; Hprt1 reverse, *cgtgattcaaatccctgaagt, GAPDH* forward, *TGAAGCAGGCATCTGAGGG*; *GAPDH* reverse *CGAAGGTGGAAGAGTGGGAG*, *RPL13a* forward *CCTGCTGCTCTCAAGGTT*; *RPL13a* reverse, *TGGTTGTCACTGCCTGGTACTT, Lyz1* forward, gccaaggtctaacaatcgttgtgagttg, Lyz1 reverse, cagtcagccagcttgacaccacg.

### Statistical analysis

All statistical analyses were performed using GraphPad Prism (versions 8 or 9), unless otherwise specified. The specific tests used are indicated in the corresponding figure legends. For most comparisons, an unpaired two-tailed Student’s *t*-test was applied. For pooled data from T-D immunization and from fecal microbiome experiments, the nonparametric Mann–Whitney *U*-test was used. In EAE experiments involving separately caged CYLD KI and WT mice, clinical scores and relative body weights were analysed as repeated measurements using two-way Repeated Measures (RM) ANOVA. For pooled EAE datasets, repeated measurements were analyzed using a linear mixed model fitted with the residual maximum likelihood (REML) approach, implemented in Genstat for Windows, 21st Edition (VSN International, Hemel Hempstead, UK; https://genstat.kb.vsni.co.uk). The model used was: response = μ +  genotype + housing + time + genotype.time + genotype.housing + housing.time + genotype.housing.time + subject.time. Here, the subject.time interaction term accounts for residual error due to correlations among repeated measurements within the same subject. Times of measurement were set at equal intervals. An autoregressive correlation structure was selected to model serial correlation in clinical scores, while an unstructured correlation model was used for relative body weight data. Significance of fixed main effects and interaction terms (two-way and three-way) was assessed using a modified *F*-test. All data were presented as mean ± standard error of the mean (SEM). A significance threshold of α = 0.05 was applied, although trends with slightly higher *p* values were also considered potentially informative (Wasserstein et al, 2019).

## Supplementary information

Peer Review File

Source data Fig. 1

Source data Fig. 2

Source data Fig. 3

Source data Fig. 4

Source data Fig. 5

Source data Fig. 6

Figure EV1 Source Data

Figure EV3 Source Data

Figure EV4 (b,c) Source Data

Expanded View Figures

## Data Availability

Source data for microscopy images produced in this study are available in the Zenodo database: https://zenodo.org/records/19709510. The 16S rRNA gene sequencing datasets generated in this study are publicly available in the NCBI BioProject database (accession number: PRJNA1444011; https://dataview.ncbi.nlm.nih.gov/object/PRJNA1444011?reviewer=iqpqsj6c2dajjb2q7jdqekl99c). The source data of this paper are collected in the following database record: biostudies:S-SCDT-10_1038-S44319-026-00814-4.

## References

[CR1] Baens M, Stirparo R, Lampi Y, Verbeke D, Vandepoel R, Cools J, Marynen P, de Bock CE, Bornschein S (2018) Malt1 self-cleavage is critical for regulatory T cell homeostasis and anti-tumor immunity in mice. Eur J Immunol 48:1728–1738 30025160 10.1002/eji.201847597 PMC6220888

[CR2] Bardet M, Unterreiner A, Malinverni C, Lafossas F, Vedrine C, Boesch D, Kolb Y, Kaiser D, Glück A, Schneider MA et al (2018) The T-cell fingerprint of MALT1 paracaspase revealed by selective inhibition. Immunol Cell Biol 96:81–99 29359407 10.1111/imcb.1018

[CR3] Bell PA, Scheuermann S, Renner F, Pan CL, Lu HY, Turvey SE, Bornancin F, Régnier CH, Overall CM (2022) Integrating knowledge of protein sequence with protein function for the prediction and validation of new MALT1 substrates. Comput Struct Biotechnol J 20:4717–4732 36147669 10.1016/j.csbj.2022.08.021 PMC9463181

[CR4] Berer K, Gerdes LA, Cekanaviciute E, Jia X, Xiao L, Xia Z, Liu C, Klotz L, Stauffer U, Baranzini SE et al (2017) Gut microbiota from multiple sclerosis patients enables spontaneous autoimmune encephalomyelitis in mice. Proc Natl Acad Sci USA 114:10719–10724 28893994 10.1073/pnas.1711233114 PMC5635914

[CR5] Berer K, Mues M, Koutrolos M, Rasbi Z, Al, Boziki M, Johner C, Wekerle H, Krishnamoorthy G (2011) Commensal microbiota and myelin autoantigen cooperate to trigger autoimmune demyelination. Nature 479:538–541 22031325 10.1038/nature10554

[CR6] Biswas S, Chalishazar A, Helou Y, DiSpirito J, DeChristopher B, Chatterjee D, Merselis L, Vincent B, Monroe JG, Rabah D et al (2022) Pharmacological inhibition of MALT1 ameliorates autoimmune pathogenesis and can be uncoupled from effects on regulatory T-cells. Front Immunol 13:875320 35615349 10.3389/fimmu.2022.875320 PMC9125252

[CR7] Biswas S, Steadman M, Helou Y, Sellers K, Soh K, Chalishazar A, Badur M, DiSpirito J, DeChristopher B, Monroe J, et al (2021) Pharmacological inhibition of MALT1 reverses activation-induced metabolic reprogramming and ameliorates autoimmune pathogenesis in multiple animal models of chronic inflammation. Arthritis Rheumatol 13:875320

[CR8] Bolyen E, Rideout JR, Dillon MR, Bokulich NA, Abnet CC, Al-Ghalith GA, Alexander H, Alm EJ, Arumugam M, Asnicar F et al (2019) Reproducible, interactive, scalable and extensible microbiome data science using QIIME 2. Nat Biotechnol 37:852–857 31341288 10.1038/s41587-019-0209-9 PMC7015180

[CR9] Bornancin F, Renner F, Touil R, Sic H, Kolb Y, Touil-Allaoui I, Rush JS, Smith PA, Bigaud M, Junker-Walker U et al (2015) Deficiency of MALT1 paracaspase activity results in unbalanced regulatory and effector T and B cell responses leading to multiorgan inflammation. J Immunol 194:3723–3734 25762782 10.4049/jimmunol.1402254

[CR10] Cheng L, Deng N, Yang N, Zhao X, Lin X (2019) Malt1 protease is critical in maintaining function of regulatory T cells and may be a therapeutic target for antitumor immunity. J Immunol 202:3008 30979818 10.4049/jimmunol.1801614

[CR11] Cosorich I, Dalla-Costa G, Sorini C, Ferrarese R, Messina MJ, Dolpady J, Radice E, Mariani A, Testoni PA, Canducci F et al (2022) High frequency of intestinal TH17 cells correlates with microbiota alterations and disease activity in multiple sclerosis. Sci Adv 3:e1700492 10.1126/sciadv.1700492 PMC5507635 28706993

[CR12] Demeyer A, Driege Y, Skordos I, Coudenys J, Lemeire K, Elewaut D, Staal J, Beyaert R (2020) Long-term MALT1 inhibition in adult mice without severe systemic autoimmunity. iScience 23:101557 33083726 10.1016/j.isci.2020.101557 PMC7522757

[CR13] Demeyer A, Skordos I, Driege Y, Kreike M, Hochepied T, Baens M, Staal J, Beyaert R (2019b) MALT1 proteolytic activity suppresses autoimmunity in a T cell intrinsic manner. Front Immunol 10:1898 31474984 10.3389/fimmu.2019.01898 PMC6702287

[CR14] Demeyer A, Van Nuffel E, Baudelet G, Driege Y, Kreike M, Muyllaert D, Staal J, Beyaert R (2019a) MALT1-deficient mice develop atopic-like dermatitis upon aging. Front Immunol 10:2330 31632405 10.3389/fimmu.2019.02330 PMC6779721

[CR15] Dumont C, Sivars U, Andreasson T, Odqvist L, Mattsson J, DeMicco A, Pardali K, Johansson G, Yrlid L, Cox RJ et al (2020) A MALT1 inhibitor suppresses human myeloid DC, effector T-cell and B-cell responses and retains Th1/regulatory T-cell homeostasis. PLoS ONE 15:e0222548 32870913 10.1371/journal.pone.0222548 PMC7462277

[CR16] Gewies A, Gorka O, Bergmann H, Pechloff K, Petermann F, Jeltsch KM, Rudelius M, Kriegsmann M, Weichert W, Horsch M et al (2014) Uncoupling Malt1 threshold function from paracaspase activity results in destructive autoimmune inflammation. Cell Rep 9:1292–1305 25456129 10.1016/j.celrep.2014.10.044

[CR17] Glöckner FO (2019) The SILVA Database Project: An ELIXIR core data resource for high-quality ribosomal RNA sequences. Biodivers Inf Sci Stand 3:e36125

[CR18] Gomez Solsona B, Schmitt A, Schulze-Osthoff K, Hailfinger S (2022) The paracaspase MALT1 in cancer. Biomedicines 10:344 35203553 10.3390/biomedicines10020344 PMC8961791

[CR19] Gourdain P, Ballerini C, Nicot AB, Carnaud C (2012) Exacerbation of experimental autoimmune encephalomyelitis in prion protein (PrPc)-null mice: evidence for a critical role of the central nervous system. J Neuroinflammation 9:25 22281016 10.1186/1742-2094-9-25 PMC3305405

[CR20] Hamp I, O’Neill TJ, Plettenburg O, Krappmann D (2021) A patent review of MALT1 inhibitors (2013-present). Expert Opin Ther Pat 31:1079–1096 10.1080/13543776.2021.1951703 34214002

[CR21] Hövelmeyer N, Wunderlich FT, Massoumi R, Jakobsen CG, Song J, Wörns MA, Merkwirth C, Kovalenko A, Aumailley M, Strand D et al (2007) Regulation of B cell homeostasis and activation by the tumor suppressor gene CYLD. J Exp Med 204:2615–2627 17923499 10.1084/jem.20070318 PMC2118471

[CR22] Jameus A, Dougherty J, Narendrula R, Levert D, Valiquette M, Pirkkanen J, Lalonde C, Bonin P, Gagnon JD, Appanna VD et al (2024) Acute impacts of ionizing radiation exposure on the gastrointestinal tract and gut microbiome in mice. Int J Mol Sci 25:3339 38542312 10.3390/ijms25063339 PMC10970505

[CR23] Jaworski M, Marsland BJ, Gehrig J, Held W, Favre S, Luther SA, Perroud M, Golshayan D, Gaide O, Thome M (2014) Malt1 protease inactivation efficiently dampens immune responses but causes spontaneous autoimmunity. EMBO J 33:2765–2781 25319413 10.15252/embj.201488987 PMC4282555

[CR24] Jeltsch KM, Hu D, Brenner S, Zöller J, Heinz GA, Nagel D, Vogel KU, Rehage N, Warth SC, Edelmann SL et al (2014) Cleavage of roquin and regnase-1 by the paracaspase MALT1 releases their cooperatively repressed targets to promote TH17 differentiation. Nat Immunol 15:1079–1089 25282160 10.1038/ni.3008

[CR25] Jin W, Reiley WR, Lee AJ, Wright A, Wu X, Zhang M, Sun S-C (2007) Deubiquitinating enzyme CYLD regulates the peripheral development and naive phenotype maintenance of B cells. J Biol Chem 282:15884–15893 17392286 10.1074/jbc.M609952200

[CR26] Juilland M, Alouche N, Ubezzi I, Gonzalez M, Rashid H-O, Scarpellino L, Erdmann T, Grau M, Lenz G, Luther SA et al (2023) Identification of Tensin-3 as a MALT1 substrate that controls B cell adhesion and lymphoma dissemination. Proc Natl Acad Sci USA 120:e2301155120 38109544 10.1073/pnas.2301155120 PMC10756297

[CR27] Juilland M, Thome M (2016) Role of the CARMA1/BCL10/MALT1 complex in lymphoid malignancies. Curr Opin Hematol 23:402–409 27135977 10.1097/MOH.0000000000000257 PMC4900422

[CR28] Klei LR, Hu D, Panek R, Alfano DN, Bridwell RE, Bailey KM, Oravecz-Wilson KI, Concel VJ, Hess EM, Van Beek M et al (2016) MALT1 protease activation triggers acute disruption of endothelial barrier integrity via CYLD cleavage. Cell Rep 17:221–232 27681433 10.1016/j.celrep.2016.08.080 PMC5087334

[CR29] Klindworth A, Pruesse E, Schweer T, Peplies J, Quast C, Horn M, Glöckner FO (2013) Evaluation of general 16S ribosomal RNA gene PCR primers for classical and next-generation sequencing-based diversity studies. Nucleic Acids Res 41:e1 22933715 10.1093/nar/gks808 PMC3592464

[CR30] Kohm AP, Carpentier PA, Anger HA, Miller SD (2002) Cutting edge: CD4+CD25+ regulatory T cells suppress antigen-specific autoreactive immune responses and central nervous system inflammation during active experimental autoimmune encephalomyelitis. J Immunol 169:4712 12391178 10.4049/jimmunol.169.9.4712

[CR31] Kong G, Dou Y, Xiao X, Wang Y, Ming Y, Li XC (2021) Transgenic expression of a mutant ribonuclease regnase-1 in T cells disturbs T cell development and functions. Front Immunol 12:682220 34305914 10.3389/fimmu.2021.682220 PMC8297167

[CR32] Kumar P, Monin L, Castillo P, Elsegeiny W, Horne W, Eddens T, Vikram A, Good M, Schoenborn AA, Bibby K et al (2016) Intestinal interleukin-17 receptor signaling mediates reciprocal control of the gut microbiota and autoimmune inflammation. Immunity 44:659–671 26982366 10.1016/j.immuni.2016.02.007 PMC4794750

[CR33] Lee JH, Zou L, Yang R, Han J, Wan Q, Zhang X, El Baghdady S, Roman A, Elly C, Jin H et al (2021) The deubiquitinase CYLD controls protective immunity against helminth infection by regulation of Treg cell plasticity. J Allergy Clin Immunol 148:209–224.e9 33309741 10.1016/j.jaci.2020.10.042 PMC8729234

[CR34] Lee P, Zhu Z, Hachmann J, Nojima T, Kitamura D, Salvesen G, Rickert RC (2017) Differing requirements for malt1 function in peripheral B cell survival and differentiation. J Immunol 198:1066 28031341 10.4049/jimmunol.1502518 PMC5263101

[CR35] Liu W, Guo W, Hang N, Yang Y, Wu X, Shen Y, Cao J, Sun Y, Xu Q (2016) MALT1 inhibitors prevent the development of DSS-induced experimental colitis in mice via inhibiting NF-κB and NLRP3 inflammasome activation. Oncotarget 7:30536–30549 27105502 10.18632/oncotarget.8867 PMC5058699

[CR36] Luo P, Yang Z, Chen B, Zhong X (2020) The multifaceted role of CARD9 in inflammatory bowel disease. J Cell Mol Med 24:34–39 31696662 10.1111/jcmm.14770 PMC6933369

[CR37] Martin F, Oliver AM, Kearney JF (2001) Marginal zone and B1 B cells unite in the early response against T-independent blood-borne particulate antigens. Immunity 14:617–629 11371363 10.1016/s1074-7613(01)00129-7

[CR38] Martin K, Junker U, Tritto E, Sutter E, Rubic-Schneider T, Morgan H, Niwa S, Li J, Schlapbach A, Walker D et al (2020) Pharmacological inhibition of MALT1 protease leads to a progressive IPEX-like pathology. Front Immunol 11:745 32425939 10.3389/fimmu.2020.00745 PMC7203682

[CR39] Mc Guire C, Elton L, Wieghofer P, Staal J, Voet S, Demeyer A, Nagel D, Krappmann D, Prinz M, Beyaert R et al (2014) Pharmacological inhibition of MALT1 protease activity protects mice in a mouse model of multiple sclerosis. J Neuroinflammation 11:124 25043939 10.1186/1742-2094-11-124 PMC4112826

[CR40] Mempel TR, Krappmann D (2022) Combining precision oncology and immunotherapy by targeting the MALT1 protease. J Immunother Cancer 10:e005442 36270731 10.1136/jitc-2022-005442 PMC9594517

[CR41] Miclotte L, De Paepe K, Rymenans L, Callewaert C, Raes J, Rajkovic A, Van Camp J, Van de Wiele T (2020) Dietary Emulsifiers alter composition and activity of the human gut microbiota in vitro, irrespective of chemical or natural emulsifier origin. Front Microbiol 11:577474 33250870 10.3389/fmicb.2020.577474 PMC7676226

[CR42] Minderman M, Lantermans HC, Grüneberg LJ, Cillessen SAGM, Bende RJ, van Noesel CJM, Kersten MJ, Pals ST, Spaargaren M (2023) MALT1-dependent cleavage of CYLD promotes NF-κB signaling and growth of aggressive B-cell receptor-dependent lymphomas. Blood Cancer J 13:37 36922488 10.1038/s41408-023-00809-7 PMC10017792

[CR43] Moud BN, Ober F, O’Neill TJ, Krappmann D (2024) MALT1 substrate cleavage: what is it good for? Front Immunol 15:1412347 38863711 10.3389/fimmu.2024.1412347 PMC11165066

[CR44] Nunettsu Asaba K, Okimura K, Adachi Y, Tokumaru K, Goto Y, Fujii S, Watanabe A, Sakai C, Sakurada E, Amikura K et al (2023) Discovery of orally bioavailable inhibitors of MALT1 with in vivo activity for psoriasis. Bioorg Med Chem Lett 82:129155 36720321 10.1016/j.bmcl.2023.129155

[CR45] Regen T, Isaac S, Amorim A, Núñez NG, Hauptmann J, Shanmugavadivu A, Klein M, Sankowski R, Mufazalov IA, Yogev N et al (2021) IL-17 controls central nervous system autoimmunity through the intestinal microbiome. Sci Immunol 6:eaaz6563 33547052 10.1126/sciimmunol.aaz6563

[CR46] Reiley WW, Jin W, Lee AJ, Wright A, Wu X, Tewalt EF, Leonard TO, Norbury CC, Fitzpatrick L, Zhang M et al (2007) Deubiquitinating enzyme CYLD negatively regulates the ubiquitin-dependent kinase Tak1 and prevents abnormal T cell responses. J Exp Med 204:1475–1485 17548520 10.1084/jem.20062694 PMC2118606

[CR47] Reiley WW, Zhang M, Jin W, Losiewicz M, Donohue KB, Norbury CC, Sun S-C (2006) Regulation of T cell development by the deubiquitinating enzyme CYLD. Nat Immunol 7:411–417 16501569 10.1038/ni1315

[CR48] Reissig S, Hövelmeyer N, Weigmann B, Nikolaev A, Kalt B, Wunderlich TF, Hahn M, Neurath MF, Waisman A (2012) The tumor suppressor CYLD controls the function of murine regulatory T cells. J Immunol 189:4770–4776 23066153 10.4049/jimmunol.1201993

[CR49] Rosenbaum M, Gewies A, Pechloff K, Heuser C, Engleitner T, Gehring T, Hartjes L, Krebs S, Krappmann D, Kriegsmann M et al (2019) Bcl10-controlled Malt1 paracaspase activity is key for the immune suppressive function of regulatory T cells. Nat Commun 10:2352 31138793 10.1038/s41467-019-10203-2 PMC6538646

[CR50] Ruefli-Brasse AA, French DM, Dixit VM (2003) Regulation of NF-κB-dependent lymphocyte activation and development by paracaspase. Science 302:1581–1584 14576442 10.1126/science.1090769

[CR51] Ruland J, Hartjes L (2019) CARD–BCL-10–MALT1 signalling in protective and pathological immunity. Nat Rev Immunol 19:118–134 30467369 10.1038/s41577-018-0087-2

[CR52] Ruland J, Duncan GS, Wakeham A, Mak TW (2003) Differential requirement for Malt1 in T and B cell antigen receptor signaling. Immunity 19:749–758 14614861 10.1016/s1074-7613(03)00293-0

[CR53] Schmidt H, Raj T, O’Neill TJ, Muschaweckh A, Giesert F, Negraschus A, Hoefig KP, Behrens G, Esser L, Baumann C et al (2023) Unrestrained cleavage of Roquin-1 by MALT1 induces spontaneous T cell activation and the development of autoimmunity. Proc Natl Acad Sci USA 120:e2309205120 37988467 10.1073/pnas.2309205120 PMC10691344

[CR54] Skordos I, Demeyer A, Beyaert R (2021) Analysis of T cells in mouse lymphoid tissue and blood with flow cytometry. STAR Protoc 2:100351 33665631 10.1016/j.xpro.2021.100351 PMC7907922

[CR55] Skordos I, Driege Y, Haegman M, Kreike M, Staal J, Demeyer A, Beyaert R (2023) Normal lymphocyte homeostasis and function in MALT1 protease-resistant HOIL-1 knock-in mice. FEBS J 290:2032–2048 36479846 10.1111/febs.16699

[CR56] Staal J, Driege Y, Bekaert T, Demeyer A, Muyllaert D, Van Damme P, Gevaert K, Beyaert R (2011) T-cell receptor-induced JNK activation requires proteolytic inactivation of CYLD by MALT1. EMBO J 30:1742–1752 21448133 10.1038/emboj.2011.85 PMC3101995

[CR57] Stephens LA, Malpass KH, Anderton SM (2009) Curing CNS autoimmune disease with myelin-reactive Foxp3+ Treg. Eur J Immunol 39:1108–1117 19350586 10.1002/eji.200839073

[CR58] Sun S-C (2010) CYLD: a tumor suppressor deubiquitinase regulating NF-κB activation and diverse biological processes. Cell Death Differ 17:25–34 19373246 10.1038/cdd.2009.43 PMC5848464

[CR59] Uehata T, Iwasaki H, Vandenbon A, Matsushita K, Hernandez-Cuellar E, Kuniyoshi K, Satoh T, Mino T, Suzuki Y, Standley DM et al (2013) Malt1-induced cleavage of regnase-1 in CD4+ helper T cells regulates immune activation. Cell 153:1036–1049 23706741 10.1016/j.cell.2013.04.034

[CR60] Van Nuffel E, Staal J, Baudelet G, Haegman M, Driege Y, Hochepied T, Afonina IS, Beyaert R (2020) MALT1 targeting suppresses CARD14-induced psoriatic dermatitis in mice. EMBO Rep 21:e49237 32343482 10.15252/embr.201949237 PMC7332803

[CR61] Vázquez-Baeza Y, Pirrung M, Gonzalez A, Knight R (2013) EMPeror: a tool for visualizing high-throughput microbial community data. Gigascience 2:2047–217X-2–16 10.1186/2047-217X-2-16 PMC4076506 24280061

[CR62] Wasserstein RL, Schirm AL, Lazar NA (2019) Moving to a World Beyond “*p* < 0.05”. Am Stat 73:1–19

[CR63] Yu JW, Hoffman S, Beal AM, Dykon A, Ringenberg MA, Hughes AC, Dare L, Anderson AD, Finger J, Kasparcova V et al (2015) MALT1 protease activity is required for innate and adaptive immune responses. PLoS ONE 10:e0127083 25965667 10.1371/journal.pone.0127083 PMC4428694

[CR64] Zhang X, Koldzic DN, Izikson L, Reddy J, Nazareno RF, Sakaguchi S, Kuchroo VK, Weiner HL (2004) IL-10 is involved in the suppression of experimental autoimmune encephalomyelitis by CD25+CD4+ regulatory T cells. Int Immunol 16:249–256 14734610 10.1093/intimm/dxh029

