# Supplementary material for: Preventing MALT1-mediated CYLD cleavage induces intestinal dysbiosis and reduces EAE severity
Source: EMBO Rep. 2026 Jun 1;27(13):3731–58. doi: 10.1038/s44319-026-00814-4 (PMC13354562; doi:10.1038/s44319-026-00814-4)
Supplement: Supplementary file 11 — Expanded View Figures [file 44319_2026_814_MOESM11_ESM.pdf]

# Expanded View Figures

**Figure EV1. Inhibition of CYLD cleavage in CYLD(R321A) splenocytes.**

(**A**) Schematic representation of the MALT1 cleavage site at R321 in mouse CYLD, leading to an N-terminal fragment (p40) and a C-terminal fragment (p70). CRISPR/Cas9 gene editing was used to generate non-cleavable CYLD(R321A) KI mice. (**B**) Immunoblot analysis of MALT1-mediated cleavage of CYLD, Regnase-1, and HOIL-1 in WT and CYLD KI splenocytes stimulated with PMA plus ionomycin (P/I) for the indicated times. Splenocytes from three mice per genotype (8–12 weeks old) were pooled. Full-length (FL) and cleaved (CL) forms of CYLD (p70), Regnase-1 (p55), and HOIL-1 (p22) are indicated by black and red arrowheads, respectively. Actin served as a loading control. All proteins shown, including the loading control, were run on the same gel. Data were representative of two experiments with similar results. Source data are available online for this figure.

 © The Author(s)

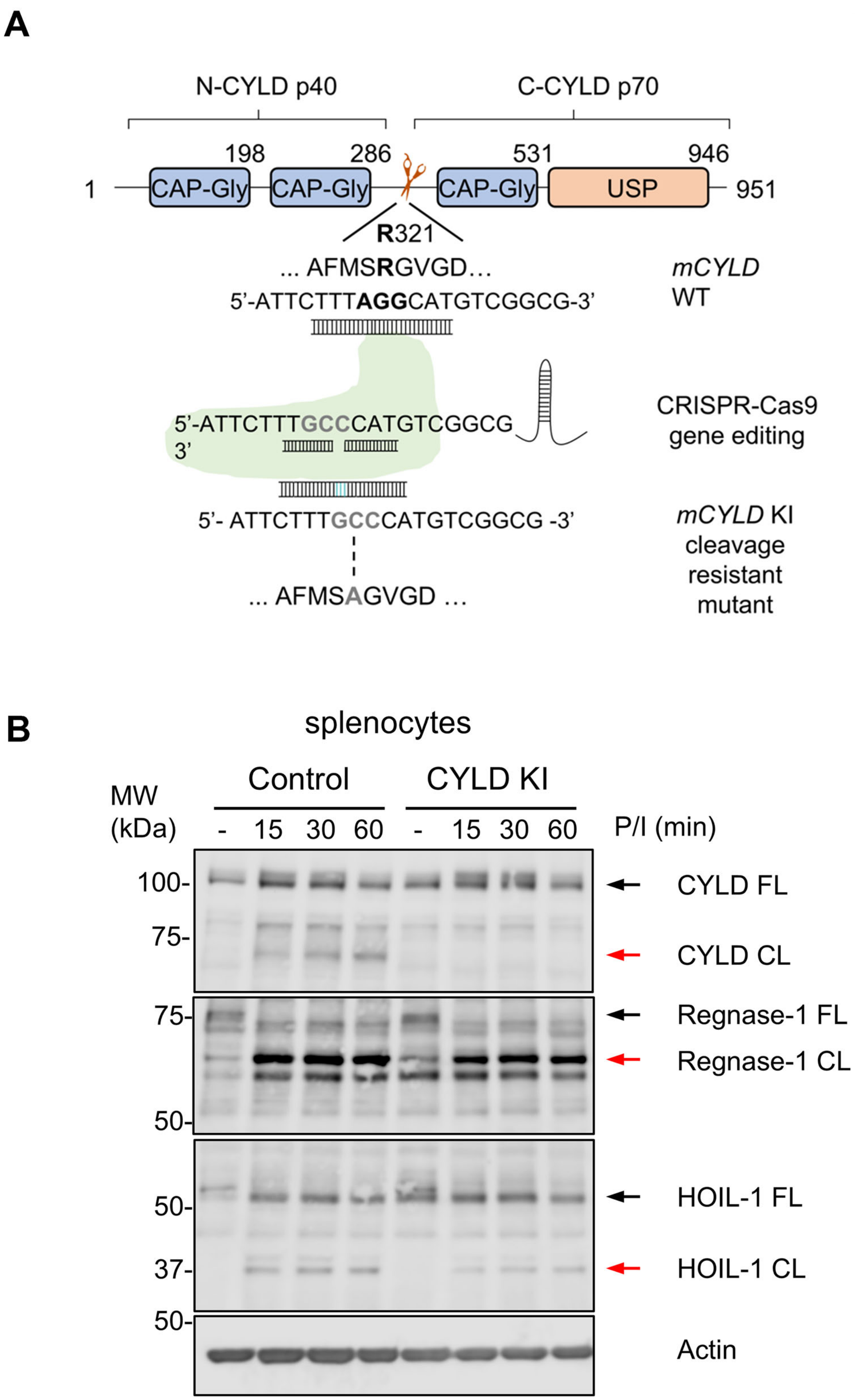
A
N-CYLD p40
C-CYLD p70
198
286
531
946
1
CAP-Gly
CAP-Gly
CAP-Gly
USP
951
R321
... AFMSRGVGD…
5'-ATTCTTTAGGCATGTCGGCG-3'
mCYLD WT
5'-ATTCTTTGCCCATGTCGGCG 3'
CRISPR-Cas9 gene editing
5'- ATTCTTTGCCCATGTCGGCG -3'
... AFMSAGVGD …
mCYLD KI cleavage resistant mutant
B
splenocytes
Control
CYLD KI
MW (kDa)
- 15 30 60 - 15 30 60
P/I (min)
100-
75-
75-
50-
50-
37-
50-
CYLD FL
CYLD CL
Regnase-1 FL
Regnase-1 CL
HOIL-1 FL
HOIL-1 CL
Actin

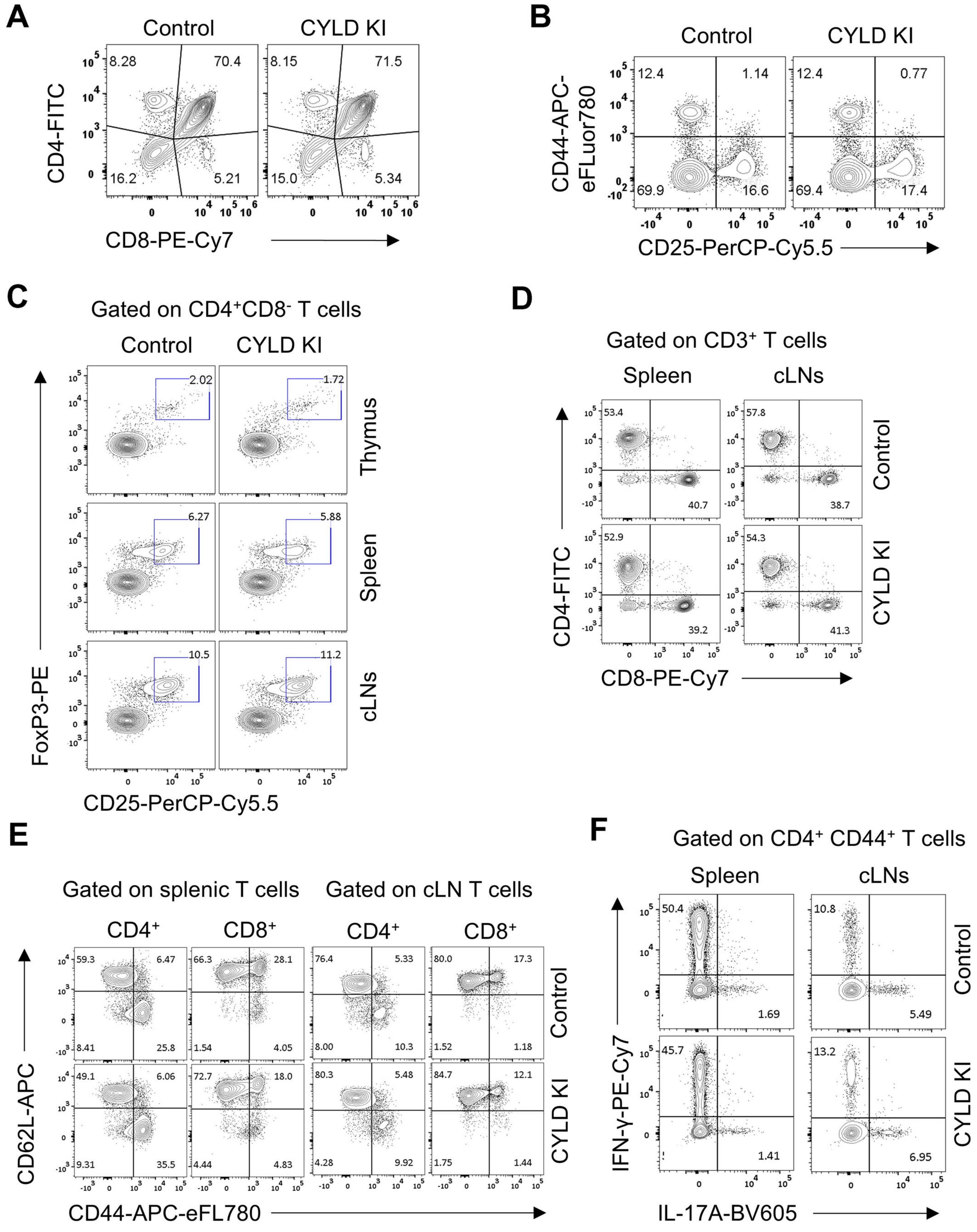
A
Control
CYLD KI
CD4-FITC
CD8-PE-Cy7
8.28
70.4
16.2
5.21
8.15
71.5
15.0
5.34
B
Control
CYLD KI
CD44-APC-eFLuor780
CD25-PerCP-Cy5.5
12.4
1.14
69.9
16.6
12.4
0.77
69.4
17.4
C
Gated on CD4+CD8- T cells
Control
CYLD KI
Thymus
Spleen
cLNs
FoxP3-PE
CD25-PerCP-Cy5.5
2.02
1.72
6.27
5.88
10.5
11.2
D
Gated on CD3+ T cells
Spleen
cLNs
Control
CYLD KI
CD4-FITC
CD8-PE-Cy7
53.4
40.7
57.8
38.7
52.9
39.2
54.3
41.3
E
Gated on splenic T cells
Gated on cLN T cells
CD4+
CD8+
CD4+
CD8+
Control
CYLD KI
CD62L-APC
CD44-APC-eFL780
59.3
6.47
8.41
25.8
66.3
28.1
1.54
4.05
76.4
5.33
8.00
10.3
80.0
17.3
1.52
1.18
49.1
6.06
9.31
35.5
72.7
18.0
4.44
4.83
80.3
5.48
4.28
9.92
84.7
12.1
1.75
1.44
F
Gated on CD4+ CD44+ T cells
Spleen
cLNs
Control
CYLD KI
IFN-γ-PE-Cy7
IL-17A-BV605
50.4
1.69
10.8
5.49
45.7
1.41
13.2
6.95

 © The Author(s)

**Figure EV2. Normal thymic and peripheral T cell compartments in CYLD KI mice.**

(**A**) Representative contour plots showing the frequencies of thymocyte subsets in CYLD KI and control WT mice (8–12 weeks old). Populations include double-negative (DN; CD4$^{-}$CD8$^{-}$CD3$^{-}$), double-positive (DP; CD4$^{+}$CD8$^{+}$CD3$^{-}$), and single-positive (SP; CD4$^{+}$CD3$^{-}$ or CD8$^{+}$CD3$^{-}$) cells. (**B**) Representative contour plots showing the distribution of DN thymocyte subsets: DN1 (CD44$^{+}$CD25$^{-}$), DN2 (CD44$^{+}$CD25$^{+}$), DN3 (CD44$^{-}$CD25$^{+}$), and DN4 (CD44$^{-}$CD25$^{-}$). (**C**) Representative contour plots showing the frequency of Tregs (CD25$^{+}$FoxP3$^{+}$) gated on CD4$^{+}$CD8$^{-}$ T cells in the thymus, and on CD3$^{+}$CD4$^{+}$CD8$^{-}$ T cells in the spleen (SPLN) and cervical lymph nodes (cLNs). (**D**) Representative contour plots showing the frequencies of CD4$^{+}$ and CD8$^{+}$ T cells within the CD3$^{+}$ T cell population in the spleen and cLNs. (**E**) Representative contour plots showing the frequencies of naïve (CD62L$^{+}$CD44$^{-}$), central memory (CD62L$^{+}$CD44$^{+}$) and effector/memory (CD62L$^{-}$CD44$^{+}$) CD4$^{+}$ and CD8$^{+}$ T cell subsets in the spleen and cLNs. (**F**) Representative contour plots of IFN-γ$^{+}$ and IL-17$^{+}$ CD44$^{+}$CD4$^{+}$ T cells from spleen and cLNs. Numbers within gates indicate the percentage of cells in each population. Data are representative of at least three experiments with similar results.

© The Author(s)

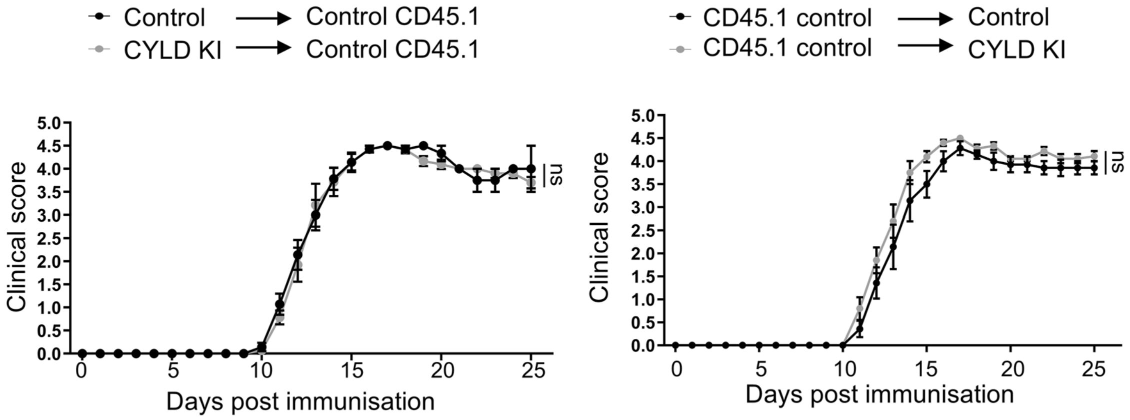


**Figure EV3. Bone marrow chimera experiments assessing the contribution of hematopoietic and non-hematopoietic compartments to EAE susceptibility.**

Clinical scores of EAE in bone marrow chimeric mice. Lethally irradiated CD45.1⁺ control (WT) recipient mice were reconstituted with $2 \times 10^6$ bone marrow cells from either CD45.2⁺ WT or CD45.2⁺ CYLD KI donors (left panel). In the reciprocal experiment, lethally irradiated CD45.2⁺ WT or CYLD KI recipient mice were reconstituted with CD45.1⁺ WT bone marrow (right panel). After 10 weeks, chimeras were immunized with $MOG_{35-55}$, and disease progression was monitored daily for 25 days. Statistical analysis: Two-way repeated measures ANOVA. Sample sizes: left panel $n = 7$ mice per group; right panel $n = 7$ WT → WT and 10 WT → CYLD KI mice. All mice were 8–14 weeks old. Data were presented as means ± SEM and are representative of two experiments with similar results. Source data are available online for this figure.

 © The Author(s)

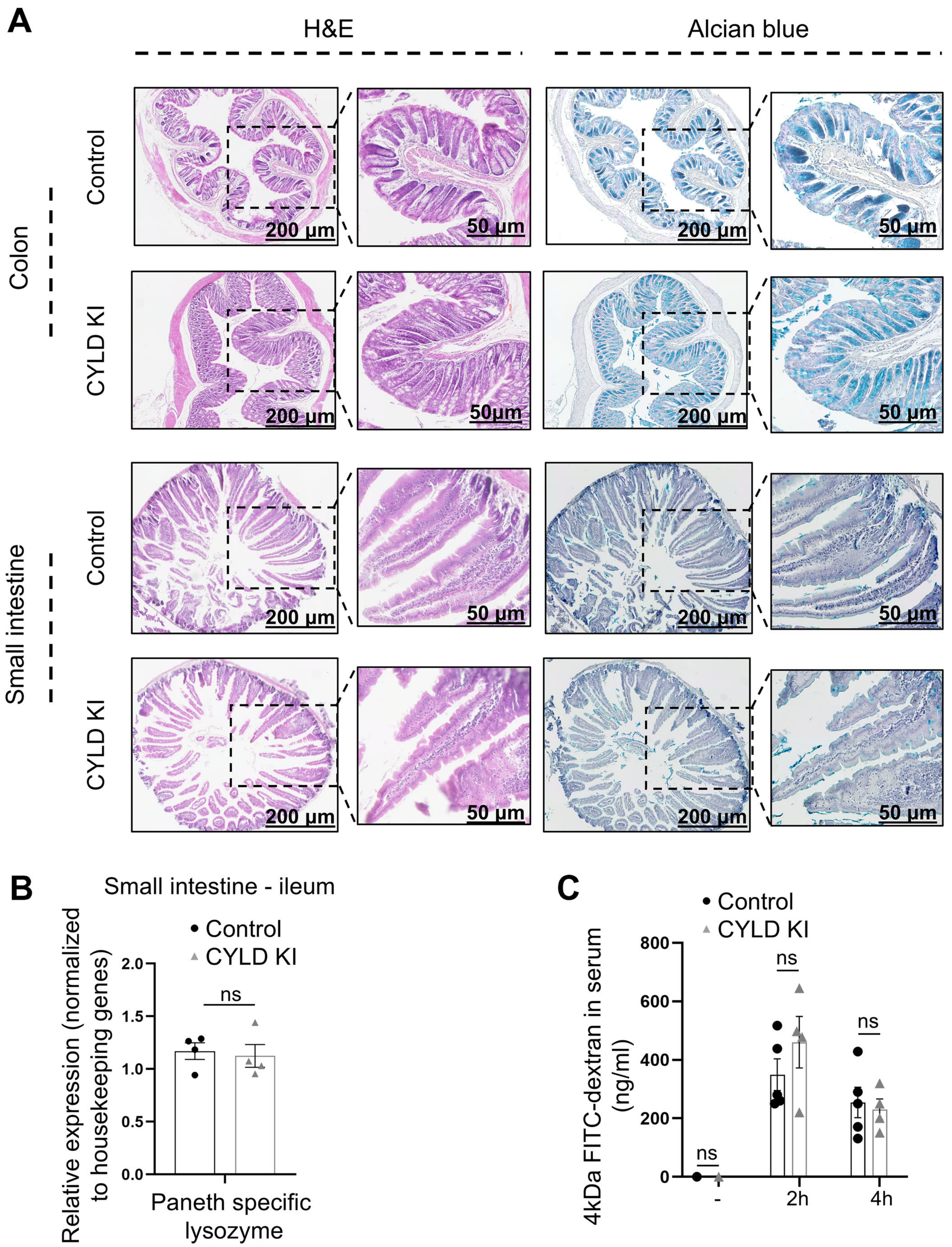
A
H&E
Alcian blue
Colon
Control
CYLD KI
Small intestine
Control
CYLD KI
200 µm
50 µm
50µm
B
Small intestine - ileum
Control
CYLD KI
Relative expression (normalized to housekeeping genes)
2.0
1.5
1.0
0.5
0.0
ns
Paneth specific lysozyme
C
Control
CYLD KI
4kDa FITC-dextran in serum (ng/ml)
800
600
400
200
0
ns
-
2h
4h

**Figure EV4. Intestinal morphology and barrier function are preserved in CYLD KI mice.**

(**A**) Representative histological sections of colon (top) and small intestine (bottom) from naïve-state WT and CYLD KI mice stained with hematoxylin and eosin (H&E, left) or Alcian Blue (right). Scale bar, 200 μm. Insets show higher magnification views. (**B**) Expression levels of the Paneth cell antimicrobial peptide lysozyme in the small intestine of WT and CYLD KI mice, determined by qPCR. (**C**) Intestinal permeability assessed by oral administration of 4-kDa FITC-dextran followed by measurement of serum fluorescence after 2 h (reflecting small intestinal permeability) and 4 h (reflecting colonic permeability). Bar graphs show mean ± SEM, with individual data points representing single animals (biological replicates). Statistical analysis was performed using a two-tailed unpaired Student's *t*-test (ns non-significant) (**B**) and mixed-effects analysis with Greenhouse–Geisser correction (ns; non-significant) (**C**). Sample sizes: (**A**) $n = 5$ WT and $n = 5$ CYLD KI mice, representative histological sections shown; (**B**) $n = 4$ WT and 4 CYLD KI mice; (**C**) $n = 5$ WT and 4 CYLD KI. All mice were 8–12 weeks old. (**B**, **C**) Data were representative of two experiments with similar results. Source data are available online for this figure.

 © The Author(s)
