# Supplementary material for: Preventing MALT1-mediated CYLD cleavage induces intestinal dysbiosis and reduces EAE severity
Source: EMBO Rep. 2026 Jun 1;27(13):3731–58. doi: 10.1038/s44319-026-00814-4 (PMC13354562; doi:10.1038/s44319-026-00814-4)
Supplement: Supplementary file 1 — Peer Review File [file 44319_2026_814_MOESM1_ESM.pdf]

EMBO reports

Preventing MALT1-mediated CYLD cleavage induces intestinal dysbiosis and reduces EAE severity

Ioannis Skordos, Elisabeth Gilis, Chris Callewaert, Aigerim Aidarova, Mira Haegman, Yasmine Driege, Marja Kreike, Inna Afonina, Jens Staal, Annelies Demeyer, Dirk Elewaut, and Rudi Beyaert

Corresponding author(s): Rudi Beyaert (Rudi.Beyaert@irc.vib-ugent.be)

Review Timeline:

Submission Date:	10th Nov 25
Editorial Decision:	8th Dec 25
Revision Received:	31st Mar 26
Editorial Decision:	17th Apr 26
Revision Received:	13th May 26
Accepted:	21st May 26

Editor: Achim Breiling

Transaction Report:

(Note: With the exception of the correction of typographical or spelling errors that could be a source of ambiguity, letters and reports are not edited. Depending on transfer agreements, referee reports obtained elsewhere may or may not be included in this compilation. Referee reports are anonymous unless the Referee chooses to sign their reports.)

Dear Prof. Beyaert,

I have already forwarded to you the reports I received from the three referees that I asked to evaluate your study. Please find them again below. After going through your preliminary point-by-points response (revision plan), I have decided to proceed with the manuscript.

Given the constructive referee comments, I would thus like to invite you to revise your manuscript with the understanding that the concerns of the referees must be addressed in the revised manuscript and/or in a detailed point-by-point response, as indicated in your revision plan. Acceptance of your manuscript will depend on a positive outcome of a second round of review, involving referees #1 and #2. It is EMBO reports policy to allow a single round of revision only and acceptance of the manuscript will therefore depend on the completeness of your responses included in the next, final version of the manuscript.

Revised manuscripts should be submitted within three months of a request for revision. Please contact me to discuss the revision (also by video chat) if you have questions or comments regarding the revision, or should you need additional time.

When submitting your revised manuscript, please also carefully review the instructions that follow below.

PLEASE NOTE THAT upon resubmission revised manuscripts are subjected to an initial quality control prior to exposition to re-review. Upon failure in the initial quality control, the manuscripts are sent back to the authors, which may lead to delays. Frequent reasons for such a failure are the lack of the data availability section (please see below) and the presence of statistics based on n=2 (the authors are then asked to present scatter plots or provide more data points).

When submitting your revised manuscript, we will require:

1) a .docx formatted version of the final manuscript text (including legends for main figures, EV figures and tables), but without the figures included. Figure legends should be compiled at the end of the manuscript text.

2) individual production quality figure files as .eps, .tif, .jpg (one file per figure), of main figures and EV figures. Please upload these as separate, individual files upon re-submission.

The Expanded View format, which will be displayed in the main HTML of the paper in a collapsible format, has replaced the Supplementary information. You can submit up to 6 images as Expanded View. Please follow the nomenclature Figure EV1, Figure EV2 etc. The figure legend for these should be included in the main manuscript document file in a section called Expanded View Figure Legends after the main Figure Legends section. Additional Supplementary material should be supplied as a single pdf file labeled Appendix. The Appendix should have page numbers and needs to include a table of content on the first page (with page numbers) and legends for all content. Please follow the nomenclature Appendix Figure Sx, Appendix Table Sx etc. throughout the text, and also label the figures and tables according to this nomenclature.

For more details, please refer to our guide to authors:
https://link.springer.com/journal/44319/submission-guidelines#cms-Revised-submissions

Please consult our guide for figure preparation:
https://media.springernature.com/original/springer-cms/rest/v1/content/27825798/data/v1

3) a .docx formatted letter INCLUDING the reviewers' reports and your detailed point-by-point responses to their comments. As part of the EMBO Press transparent editorial process, the point-by-point response is part of the Review Process File (RPF), which will be published alongside your paper.

4) a complete author checklist, which you can download from our author guidelines (https://link.springer.com/journal/44319/submission-guidelines#cms-Revised-submissions). Please insert page numbers in the checklist to indicate where the requested information can be found in the manuscript. The completed author checklist will also be part of the RPF.

Please also follow our guidelines for the use of living organisms, and the respective reporting guidelines:
https://link.springer.com/partners/embo-press/editorial-policies#Animal%20and%20human%20studies

5) that primary datasets produced in this study (e.g. RNA-seq, ChIP-seq, structural and array data) are deposited in an appropriate public database. If no primary datasets have been deposited, please also state this in a dedicated section (e.g. 'No primary datasets have been generated and deposited'), see below.

See also: https://link.springer.com/partners/embo-press/editorial-policies#Data%20deposition

Please remember to provide a reviewer password if the datasets are not yet public.

The accession numbers and database should be listed in a formal "Data Availability" section that follows the model below. This is now mandatory (like the COI statement). Please note that the Data Availability Section is restricted to new primary data that are part of this study. This section is mandatory. As indicated above, if no primary datasets have been deposited, please state this in this section

Data availability

The datasets produced in this study are available in the following databases:

- RNA-Seq data: Gene Expression Omnibus GSE46843 (https://www.ncbi.nlm.nih.gov/geo/query/acc.cgi?acc=GSE46843)
- [data type]: [name of the resource] [accession number/identifier/doi] ([URL or identifiers.org/DATABASE:ACCESSION])

*** Note - All links should resolve to a page where the data can be accessed. ***

See also: https://link.springer.com/partners/embo-press/editorial-policies#Data%20availability%20statement

Moreover, I have these editorial requests:

6) We now request the publication of original source data with the aim of making primary data more accessible and transparent to the reader. You will receive a separate email with instructions for providing source data with your revised manuscript, including information how to upload and organize the files.

7) Our journal encourages inclusion of *data citations in the reference list* to directly cite datasets that were re-used and obtained from public databases. Data citations in the article text are distinct from normal bibliographical citations and should directly link to the database records from which the data can be accessed. In the main text, data citations are formatted as follows: "Data ref: Smith et al, 2001" or "Data ref: NCBI Sequence Read Archive PRJNA342805, 2017". In the Reference list, data citations must be labeled with "[DATASET]". A data reference must provide the database name, accession number/identifiers and a resolvable link to the landing page from which the data can be accessed at the end of the reference. Further instructions are available at: https://link.springer.com/journal/44319/submission-guidelines#cms-Reference-guidelines

8) Regarding data quantification and statistics, please make sure that the number "n" for how many independent experiments were performed, their nature (biological versus technical replicates), the bars and error bars (e.g. SEM, SD) and the test used to calculate p-values is indicated in the respective figure legends (also for EV and Appendix figures). Please also check that all the p-values are explained in the legend, and that these fit to those shown in the figure. Please provide statistical testing where applicable. Please avoid the phrase 'independent experiment' but clearly state if these were biological or technical replicates. Please also indicate (e.g. with n.s.) if testing was performed, but the differences are not significant. In case n=2, please show the data as separate datapoints without error bars and statistics. See also:
https://link.springer.com/journal/44319/submission-guidelines#cms-Figure-and-data-presentation

If n<5, please show single datapoints for diagrams.

9) Please confirm that for all Western blot panels shown the loading control was run on the same gel as the other proteins detected. Please note that we discourage comparisons between samples on different gels/blots, even if the samples derive from one experiment, as confounding factors reduce comparability. If unavoidable, the figure legend must state that the samples derive from the same experiment and that gels/blots were processed in parallel. If a 'representative' loading control is shown for multiple gels/blots, the intra-gel controls should be shown in the source data files, and the figure legends should describe the data displayed accurately. See our author guidelines:

https://link.springer.com/journal/44319/submission-guidelines#cms-Figure-and-data-presentation (section 'Electrophoretic gels and blots').

10) Please also note our reference format:
https://link.springer.com/journal/44319/submission-guidelines#cms-Reference-guidelines

11) We updated our journal's competing interests policy in January 2022 and request authors to consider both actual and perceived competing interests. Please review the policy https://www.embopress.org/competing-interests and update your competing interests if necessary. Please name this section 'Disclosure and Competing Interests Statement' and put it after the Acknowledgements section.
See: https://link.springer.com/partners/embo-press/editorial-policies#Competing%20interest%20disclosures

12) We now use CRediT to specify the contributions of each author in the journal submission system. CRediT replaces the author contribution section. Please use the free text box to provide more detailed descriptions and do NOT provide your final

manuscript text file with an author contributions section. See also our guide to authors (section 'Author contributions'): https://link.springer.com/journal/44319/submission-guidelines#cms-Revised-submissions

13) All Materials and Methods need to be described in the main text using our 'Structured Methods' format, which is required for all research articles. According to this format, the Methods section should include a Reagents and Tools Table (listing key reagents, experimental models, software, and relevant equipment and including their sources and relevant identifiers), uploaded as separate file, and a Methods section in which we encourage the authors to describe their methods using a step-by-step protocol format with bullet points, to facilitate the adoption of the methodologies across labs. More information on how to adhere to this format as well as downloadable templates (.doc) for the Reagents and Tools Table can be found in our author guidelines (section 'Structured Methods'):

https://link.springer.com/journal/44319/submission-guidelines#cms-Manuscript-organisation-and-formatting

14) Please order the manuscript sections like this, using only these names:
Title page - Abstract (max. 175 words) - Keywords - Introduction - Results - Discussion - Methods - Data availability section - Acknowledgements (please include here all the funding information) - Disclosure and Competing Interests Statement - References - Figure legends - Expanded View Figure legends

15) Please make sure that all the funding information is also entered into the online submission system and that it is complete and similar to the one in the acknowledgement section of the manuscript text file.

We would also welcome the submission of cover suggestions, or motifs to be used by our Graphics Illustrator in designing a cover.

As part of the EMBO publication's Transparent Editorial Process, EMBO reports publishes online a Review Process File (RPF) to accompany accepted manuscripts. This File will be published in conjunction with your paper and will include the referee reports, your point-by-point response and all pertinent correspondence relating to the manuscript.

You are able to opt out of this by letting the editorial office know (emboreports@embo.org). If you do opt out, the Review Process File link will point to the following statement: "No Review Process File is available with this article, as the authors have chosen not to make the review process public in this case."

I look forward to seeing a revised form of your manuscript when it is ready.

Yours sincerely,

Achim Breiling
Senior Editor
EMBO Reports

Referee #1:

The manuscript by Skordos et al. addresses the relevance of CYLD cleavage in vivo through a knock-in mouse model expressing a MALT1-cleavage resistant CYLD. The authors started from a clear hypothesis, based on the autoinflammatory syndrome phenotype observed in the MALT1 protease-dead knock-in mice. Since the substrate responsible for the autoimmune phenotype remains unknown, in this manuscript the authors addressed the role of CYLD cleavage in that process. The findings are interesting, especially for clarifying that the role of CYLD cleavage observed in vitro is different than the one observed in vivo in mice: in the literature, overexpression of non-cleavable CYLD in cell lines shows a role in modulation of NF-kB and JNK signaling, however, this study shows that in vivo there is no such alteration. Also, although there was no difference in lymphocyte development, activation and differentiation in non-cleavable CYLD mice under steady state conditions, WT and CYLD KI mice showed different responses to MOG immunization, in an EAE model of multiple sclerosis. However, these differences were lost when the mice were co-housed, suggesting an important role for the microbiome/housing conditions (rather than the genotype of the mice) in the observed phenotype. Therefore, I think that at this point, more data are needed to support the proposed role of CYLD cleavage in EAE severity.

Major concerns:

1) Analysis of the EAE score for the WT mice in the two different mouse houses should be assessed and compared to CYLD KI mice that are separately housed or co-housed with WT mice in each of the two mouse facilities. At this point it cannot be excluded that the differences seen are simply due to a different microbiome in the two mouse houses (including a potential effect on the hematopoietic cells used for the BM reconstitution, which came from WT mice from two different mouse houses) rather

than due to genetic differences.

2) The observed differences in the microbiome of wt and CylD KI mice under conditions of separate housing are not very significant, and most of all, not meaningful because the experiment is comparing two different mouse strains in two different mouse houses. Have the authors compared the microbiome of WT mice housed in the two different places? And of WT versus KI mice that are co-housed?

3) If the working hypothesis (that non-cleavable CylD affects the gut microbiome) can be proven, then it should be further explored how CYLD affects the microbiota. It was shown by Linda R Klei et al (2016) that MALT1 protease activity disrupts the endothelial barrier through CYLD cleavage. Could this be related to the authors' observations? Could the authors check the state of the gut epithelial lining in control and CYLD-KI by histology? And explore potential alterations in tight junctions?

Minor points:

1) A graphical abstract would be helpful to summarize the findings; this would be especially useful if there was a hint on the mechanism leading to reduced EAE susceptibility in separately housed CYLD KI mice.

2) In the second sentence of the last paragraph in the introduction the word "immune" is written twice.

3) In Fig. 1b, cleavage of Regnase-1 and Hoil-1 are not very well visible from that blot.

4) Overall, the structure of the manuscript could be improved and the (mainly negative data) shown in Fig. 1-6 could be grouped into 2 figures: Fig. 1 and 2 could be grouped into one figure, and Fig. 3-6 into another figure (keeping only graphs with quantification in the main figures and contour plots in supplemental material)

5) Y-axis of the flow cytometry data in Fig. 8c needs to be labelled.

6) In the contour plots of Fig. 4C, 5A, please identify the control and CYLD KI.

7) In Fig. 7A, indicate in the figure that the data is from the spleen (everywhere else it is specified).

8) In the display of Fig. 7B, it is not clear that the contour plot is part of 7B (it seems to be more aligned with Fig. 7C). Maybe swap that contour plot with the actual Fig. 7C to make it easier to the reader (or move it to the supplement).

9) Fig. 7C and 7D: please explain in the legend the meaning of TI-2 and T-D.

10) In Fig. 10A and 10B, please include in the figure panel a legend for the lighter blue and darker blue dots.

11) The size and type of the font of the letters used to annotate the figures varies greatly, please be consistent throughout the figures

----
Referee #2:

Review of MS #EMBOR-2025-63159V1 "Blocking MALT1-mediated CYLD cleavage alters gut microbiota and EAE severity without impairing lymphocyte function" by Skordos et al.
The authors present a novel mouse model in which Cyld is mutated to become resistant to MALT1 paracaspase cleavage. The authors go through a large number of experiments yielding negative results to show that in the absence of CYLD cleavage:
• JNK and IKK signaling is unaffected in T and B cells
• T cell proliferation is unchanged
• T cell activation and IL-2 production is unaltered
• thymocyte development is normal
• Treg cell frequencies are unaffected and phenotypically normal
• CD4 and CD8 Tcon frequencies and phenotypes and Th1 and Th17 effector differentiation are not altered
• B cell homeostasis and T-independent and T-dependent antibody induction are normal.
• Finally, they find that mice with blocked CYLD cleavage are partially protected from EAE, a phenotype that was due to the cohousing of the Knockin mice, and was caused by an altered microbiome.
In principle, I find it important that also negative data are reported, and the presented data are sound. However, the manuscript gives the rather unbalanced impression that CYLD cleavage has no role in lymphocytes, and also HOIL and Roquin-1 cleavage do not recapitulate the autoinflammatory MALT1 PD phenotype and therefore they suggest Regnase-1 as the main and only critical target of MALT1 paracaspase. Previous work on uncleavable Roquin-1 (with a redundant paralog Roquin-2 and the cooperating factor Regnase-1) has for example shown that these mice have reduced thymic Treg cells, however peripheral Tregs are normal. Furthermore, in these mice it was necessary to involve constitutively active MALT1 paracaspase function to

uncover critical contributions, which is not mentioned or discussed.

Major point 1. I find it important to experimentally demonstrate that uncleavable CYLD is actually a gain-of-function version of the wild-type protein.

Major point 2. The authors should consider/discuss whether the autoinflammatory MALT1 paracaspase-dead phenotype could also arise due to counter-regulation /negative feedback, since also constitutively active MALT1 induces a lethal autoinflammatory phenotype.

Major point 3. The statement .... Roquin-1 does not affect thymic Treg development is wrong, there was a significant reduction in the thymus....

Major point 4. I also don't agree with the claim: ".... blocking a single MALT1 substrate is sufficient to modulate microbiota and neuroinflammation without impairing immune function..." the negative data in this manuscript do not rule out that immune functions are altered during EAE - they also do not establish that all lymphocyte functions that contribute to EAE are equal. Experiments that rule out lymphocyte contributions would need to involve genetics, i.e. to combine conditional alleles of Cyld with the Knockin allele and utilize lymphocyte specific Cre drivers.

Referee #3:

In this manuscript, Skordos et al. generate CYLD(R321A) knock-in mice expressing a MALT1-cleavage-resistant form of CYLD. The authors report that, unlike MALT1 Protease-Dead (PD) animals, CYLD KI mice are viable, exhibit normal lymphocyte development, intact immune signaling, and lack spontaneous inflammatory phenotypes. Notably, in the experimental autoimmune encephalomyelitis (EAE) model in which MALT1 PD animals are known to be protected, CYLD KI animals show reduced disease severity relative to wild-type controls. This reduced susceptibility is lost upon co-housing, pointing toward a microbiota-dependent effect. Consistently, authors find that CYLD KI animals exhibit altered gut microbial composition, including changes in the Firmicutes/Bacteroidetes ratio.

The generation of the CYLD(R321A) knock-in mouse model provides a valuable tool to address the long-standing question of whether CYLD cleavage by MALT1 influences JNK and NF-κB signaling.

The authors convincingly demonstrate that CYLD KI animals exhibit normal lymphocyte development, preserved immune responses, and no signs of spontaneous inflammation, supporting a minimal requirement for CYLD cleavage under homeostatic conditions. However, while these baseline characterizations are important, they could be more appropriately presented as supplementary material, allowing the main text to focus more directly on the central and most compelling aspect of the study, the distinct phenotype of CYLD KI mice under EAE challenge.

The microbiota-associated phenotype is intriguing and potentially impactful. However, in the absence of mechanistic insight linking CYLD cleavage to microbial composition and disease outcome, it is hard to enthusiastically endorse this manuscript for publication.

We thank the referees for their careful evaluation of the manuscript and their constructive comments, which helped us to improve the clarity of the manuscript and strengthen the mechanistic discussion. Below we provide a point-by-point response to each of the comments that were raised.

Response to Referee #1

Major Comment 1

Analysis of the EAE score for the WT mice in the two different mouse houses should be assessed and compared to CYLD KI mice that are separately housed or co-housed with WT mice in each of the two mouse facilities. At this point it cannot be excluded that the differences seen are simply due to a different microbiome in the two mouse houses (including a potential effect on the hermatopoietic cells used for the BM reconstitution, which came from WT mice from two different mouse houses) rather than due to genetic differences.

Re: We apologize for the confusion caused by the wording we used in the manuscript. The terms ‘separately housed’ and ‘co-housed’ refer to mice kept in different cages versus mice kept in the same cage, respectively, but both in the same SPF mouse facility room. This is clarified in the ‘Results’ and ‘Material and Methods’ section of the revised manuscript. We hope this clarifies things and believe that this makes the reviewer’s comment no longer relevant.

Major Comment 2

The observed differences in the microbiome of wt and CylD KI mice under conditions of separate housing are not very significant, and most of all, not meaningful because the experiment is comparing two different mouse strains in two different mouse houses. Have the authors compared the microbiome of WT mice housed in the two different places? And of WT versus KI mice that are co-housed?

Re: As explained above, all animals were maintained in the same SPF facility. Therefore, the observed microbiota differences cannot be attributed to differences between mouse facilities. The key comparison concerns mice housed in ‘separate cages’ versus mice ‘co-housed’ in the same cage, which allows microbial exchange.

Major Comment 3

If the working hypothesis (that non-cleavable CylD affects the gut microbiome) can be proven, then it should be further explored how CYLD affects the microbiota. It was shown by Linda R Klei et al (2016) that MALT1 protease activity disrupts the endothelial barrier through CYLD cleavage. Could this be related to the authors' observations? Could the authors check the state of the gut epithelial lining in control and CYLD-KI by histology? And explore potential alterations in tight junctions?

Re: The reviewer raised an important point regarding possible changes in intestinal epithelial barrier integrity in CYLD-KI mice. To address this possibility, we performed several additional experiments, of which the results have been included in the revised manuscript as Fig. EV4.

We subjected intestinal tissue samples to histological analysis: H&E stained sections of small intestine and colon showed that there are no differences in epithelial architecture between WT and CYLD-KI mice. Similarly, Alcian Blue staining of mucins showed that goblet cell distribution was unaffected in CYLD-KI mice (Fig. EV4a).

To investigate intestinal barrier integrity, we performed an FITC–dextran permeability assay: Naïve WT and CYLD KI mice were gavaged with 4 kDa FITC–dextran, and serum fluorescence was measured at 2 and 4 h, indicating large and small intestinal permeability, respectively. No differences were observed between both genotypes (Fig. EV4b).

Finally, because Paneth cell-derived lysozyme (an antimicrobial peptide) is known to shape the composition of the intestinal microbiome and impacts intestinal homeostasis, we also analyzed its expression in the small intestine of WT and CYLD-KI mice, but no differences between both genotypes could be observed (Fig. EV4c).

Although we have not investigated any alterations in tight junctions, the above data indicate that in non-inflammatory conditions the intestinal barrier morphology and permeability of CYLD KI mice is similar to that of control mice.

Minor Comments

1) A graphical abstract would be helpful to summarize the findings; this would be especially useful if there was a hint on the mechanism leading to reduced EAE susceptibility in separately housed CYLD KI mice.

Re: This is indeed a good suggestion. We have now added a graphic abstract to the revised manuscript.

2) In the second sentence of the last paragraph in the introduction the word "immune" is written twice.

Re: We have adapted the text accordingly.

3) In Fig. 1b, cleavage of Regnase-1 and Hoil-1 are not very well visible from that blot.

Re: We apologize for the poor quality of these blots. We have now replaced them with the blots from a repeat experiment (Fig. EV1B).

4) Overall, the structure of the manuscript could be improved and the (mainly negative data) shown in Fig. 1-6 could be grouped into 2 figures: Fig. 1 and 2 could be grouped into one figure , and Fig. 3-6 into another figure (keeping only graphs with quantification in the main figures and contour plots in supplemental material).

Re: Thanks a lot for the suggestion. We adapted the presentation of the data accordingly: the former Fig. 1 is now Fig. EV1, while Fig. 2 became Fig. 1; quantification data from former figures 3–5 were combined into one figure (Fig. 2); contour plots from former figures 3-5 were grouped and moved to Fig. EV2.

5) Y-axis of the flow cytometry data in Fig. 8c needs to be labelled.

Re: We have corrected our mistake (Fig 4C; former Fig. 8C) in the revised version of our manuscript.

6) In the contour plots of Fig. 4C, 5A, please identify the control and CYLD KI.

Re: The figures were revised accordingly. We have decided to exclude the contour plot of Fig. 4C from the revised version of our manuscript. We have labelled the contour plot of Fig. 5A (= Fig. EV2D in the revised manuscript).

7) In Fig. 7A, indicate in the figure that the data is from the spleen (everywhere else it is specified).

Re: Fig. 7A (Fig. 3A in the revised version) was labeled accordingly.

8) In the display of Fig. 7B, it is not clear that the contour plot is part of 7B (it seems to be more aligned with Fig. 7C). Maybe swap that contour plot with the actual Fig. 7C to make it easier to the reader (or move it to the supplement).

Re: The figure layout (Fig. 3C in revised manuscript) has been improved. Moreover, we also modified the order of panels Fig. 7C and 7D (Fig. 3D and 3E in the revised manuscript).

9) Fig. 7C and 7D: please explain in the legend the meaning of TI-2 and T-D.

Re: Definitions have been added to the figure legends (Fig. 3D and 3E in the revised version).

10) In Fig. 10A and 10B, please include in the figure panel a legend for the lighter blue and darker blue dots.

Re: In Fig. 6A and 6B and the corresponding figure legend of the adapted version of our manuscript, we have now indicated that the dark blue and light blue dots represent mice from the first and second experiment (repeat of the first), respectively.

11) The size and type of the font of the letters used to annotate the figures varies greatly, please be consistent throughout the figures

Re: All figures were reformatted for consistency.

Response to Referee #2

In principle, I find it important that also negative data are reported, and the presented data are sound. However, the manuscript gives the rather unbalanced impression that CYLD cleavage has no role in lymphocytes, and also HOIL and Roquin-1 cleavage do not recapitulate the autoinflammatory MALT1 PD phenotype and therefore they suggest Regnase-1 as the main and only critical target of MALT1 paracaspase. Previous work on uncleavable Roquin-1 (with a redundant paralog Roquin-2 and the cooperating factor Regnase-1) has for example shown that these mice have reduced thymic Treg cells, however peripheral Tregs are normal. Furthermore, in these mice it was necessary to involve constitutively active MALT1 paracaspase function to uncover critical contributions, which is not mentioned or discussed.

Re: We apologize for not addressing in sufficient detail the immune phenotype of mice expressing other uncleavable MALT1 substrates. As also mentioned below in reply to some of the specific comments, this has now been more carefully addressed in the discussion of the revised manuscript.

Major Comment 1

I find it important to experimentally demonstrate that uncleavable CYLD is actually a gain-of-function version of the wild-type protein.

Re: We thank the reviewer for raising the important question of whether uncleavable CYLD could represent a gain-of-function variant. MALT1 cleaves mouse CYLD at R321, separating the two N-terminal microtubule-binding CAP-Gly domains from the more C-terminal third CAP-Gly domain and catalytic USP (ubiquitin-specific protease) domain. We have previously shown that both cleavage fragments are stable and accumulate over time in normal lymphocytes. Early studies describing the NF-kB inhibitory role of CYLD (PMID: 12917690; PMID: 12917689; PMID: 12917691) showed that the C-terminal USP domain and third CAP-Gly domain mediate CYLD's NF-kB inhibitory function, which is consistent with our finding that inducible NF-kB activation is similar in wild-type and uncleavable CYLD-KI cells. The first two CAP-Gly motifs (corresponding to the N-terminal cleavage fragment) have previously been shown to

associate with α-tubulin and the catalytic site of HDAC6 (a major tubulin deacetylase). This interaction inhibits HDAC6-mediated α-tubulin deacetylation, increasing the levels of acetylated α-tubulin in the cell (PMID: 19893491). While CYLD's microtubule-related functions are therefore primarily mediated by its first two CAP-Gly domains, the USP domain can influence tubulin acetylation and microtubule stability by deubiquitinating HDAC6. In this context, non-cleavable CYLD has also been shown to act as a dominant-negative to block thrombin-induced α-tubulin deacetylation in endothelial cells (PMID: 27681433). Based on these data and in response to the reviewer's question to demonstrate a possible gain-of-function effect of non-cleavable CYLD, we analyzed if preventing MALT1-mediated CYLD cleavage would affect α-tubulin acetylation in activated T cells. However, α-tubulin acetylation was found to be comparable in WT and CYLD-KI splenocytes or $CD3^+$ T cells stimulated with PMA/Iono (Experiment 1 and 2 below). Also stimulation of T cells with anti-CD3/CD28 over a more prolonged time period (similar to PMID: 16738306) did not show any differences between both genotypes (Experiment 3 below). Robust increases upon Tubastatin A (a HDAC6 inhibitor) treatment confirmed assay sensitivity across genotypes. In order not to overload the paper with more negative data, we did not include them in the revised paper but just provide them here as information for the reviewer. However, if the reviewer would find it better to also include these data in the paper, we are happy to do so.

Together, our results rule out a gain-of-function effect of non-cleavable CYLD on tubulin acetylation (and by extension microtubule regulation). We cannot exclude that the CAP-Gly domains in CYLD have any additional function, besides tubulin binding, that may be affected by CYLD cleavage.

Exp1

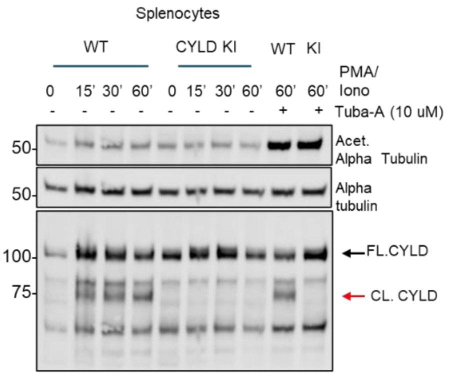

Exp2

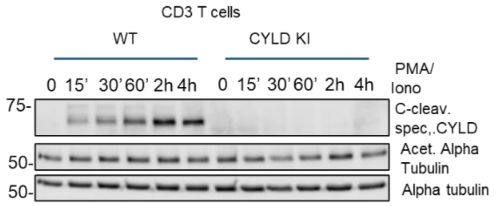

Exp3

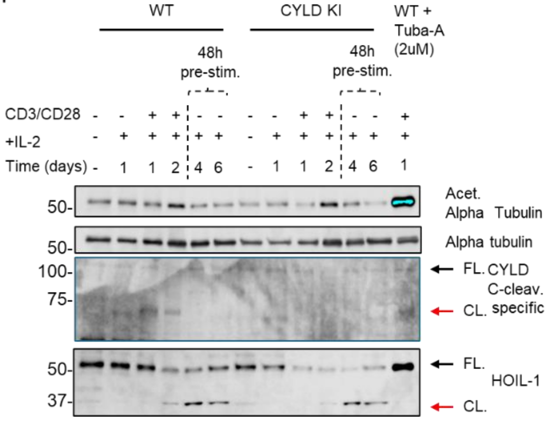

Figure legend: CYLD cleavage by MALT1 does not affect α-tubulin acetylation in lymphocytes. (**Exp1**) Splenocytes from WT and CYLD KI mice (pooled from three mice per group) were stimulated with PMA plus ionomycin (PMA/Iono) for the indicated times in the presence or absence of Tubastatin A (Tuba-A, 10 μM) as indicated. Cell lysates were analyzed by immunoblotting for acetylated α-tubulin and MALT1-mediated CYLD cleavage. Total α-tubulin served as a loading control. Full-length (FL) and cleaved (CL; p70) CYLD are indicated by black and red arrows, respectively. (**Exp2**) Purified splenic $CD3^+$ T cells from WT and CYLD KI mice (4 mice per group) were stimulated with PMA/Iono for the indicated times. Cell lysates were analyzed by immunoblotting for acetylated α-tubulin. Total α-tubulin served as a loading control. CYLD cleavage was detected using an antibody specific for the MALT1-generated C-terminal CYLD fragment (p70). (**Exp3**) Purified splenic $CD3^+$ T cells from WT and CYLD KI mice (4 mice per group) were stimulated with plate-bound anti-CD3 (2 μg/ml) and soluble anti-CD28 (2 μg/ml) in the presence or absence of Tubastatin A (Tuba-A, 2 μM) and recombinant mouse IL-2 (200 U/ml) as indicated. After 2 days, cell medium was refreshed and cells were cultured for an additional 4 days in the presence of IL-2 alone. Cell lysates collected at the indicated time points were analyzed by immunoblotting for acetylated α-tubulin, CYLD cleavage (p70), and HOIL-1 cleavage (p37). Total α-tubulin served as a loading control. Full-length (FL) and cleaved (CL) forms of CYLD and HOIL-1 are indicated by black and red arrows.

Major Comment 2:

The authors should consider/discuss whether the autoinflammatory MALT1 paracaspase-dead phenotype could also arise due to counter-regulation /negative feedback, since also constitutively active MALT1 induces a lethal autoinflammatory phenotype.

Re: We agree with this possibility and have now expanded the discussion of the revised manuscript to address this point.

Major comment 3:

The statement .... Roquin-1 does not affect thymic Treg development is wrong, there was a significant reduction in the thymus....

Re: We thank the reviewer for raising this point and apologize for mentioning it wrongly. We agree that a small reduction in the frequency of thymic Treg cells (CD4 single-positive $Foxp3^+$) has been reported for *Rc3h1^Mins/Mins* mice. However, this reduction is limited to frequencies and does not translate into a decrease in absolute thymic Treg numbers, because total thymocyte numbers are slightly increased in these mice. To avoid confusion, we have changed the wording in the revised manuscript to clarify that preventing Roquin-1 cleavage does not result in major defects in thymic Treg development.

Major comment 4:

I also don't agree with the claim: ".... blocking a single MALT1 substrate is sufficient to modulate microbiota and neuroinflammation without impairing immune function..." the negative data in this manuscript do not rule out that immune functions are altered during EAE - they also do not establish that all lymphocyte functions that contribute to EAE are equal. Experiments that rule out lymphocyte contributions would need to involve genetics, i.e. to combine conditional alleles of Cyld with the Knockin allele and utilize lymphocyte specific Cre drivers.

Re: The reviewer is correct. Absence of evidence is not the same as evidence of absence. We have now taken this along in the discussion of our revised manuscript. We assume that the referee does not want us to make the suggested conditional KI mice as this would take us at least another year. The text now states that blocking MALT1-mediated CYLD cleavage modulates microbiota composition and EAE susceptibility without causing overt defects in lymphocyte development or activation under the conditions tested. To further address the reviewer's concern, we have now also included additional experiments using bone marrow chimeras, in which lethally irradiated WT mice were reconstituted with bone marrow from either WT or CYLD KI donor mice (Fig. EV3). In this setting, EAE disease severity was comparable between groups, suggesting that the hematopoietic compartment alone is not sufficient to explain the protective phenotype observed in CYLD KI mice.

Response to Referee #3

Re: We are happy to read that this reviewer concludes that the generation of the CYLD(R321A) knock-in mouse model provides a valuable tool to address the long-standing question of whether CYLD cleavage by MALT1 influences JNK and NF-κB signaling. The reviewer also stresses that we convincingly demonstrate that CYLD KI animals exhibit normal lymphocyte development, preserved immune responses, and no signs of spontaneous inflammation, supporting a minimal requirement for CYLD cleavage under homeostatic conditions, which the reviewer also finds important. The reviewer suggested that these data could be be more appropriately presented as supplementary material. Also reviewer 1 suggested some similar reshuflling to reduce the number of figures with negative data. In response, we have now moved some of the data to Expanded View figures.

The reviewer also finds the microbiota-associated phenotype intriguing and potentially impactful, but emphasizes the absence of mechanistic insight linking CYLD cleavage to microbial composition and disease outcome. We agree with the referee's conclusion that our findings are still incomplete with respect to the underlying mechanisms for the observed link between CYLD cleavage and microbial composition and EAE disease outcome. However, I am afraid that pinpointing the exact mechanism will be very challenging and is beyond the scope of the current study. At this stage, we can only exclude a number of possibilities (like effects on epithelial barrier integrity or the role of both hematopoietic and non-hematopoietic cells).

Being aware of these limitations, the reason why we think our study might still be interesting enough to publish in EMBO Reports is because it has two very important messages that are of high interest to the field:

1. Preventing MALT1-mediated CYLD cleavage in mice does not have any impact on lymphocyte development and function, nor does it mimic the effects of protease-dead MALT1 knockin mice, which is rather unexpected based on previously published data;

2. The fact that inhibition of MALT1-mediated CYLD cleavage decreases EAE disease, without any of the severe side effects that were previously observed in the case of MALT1 protease-dead knockin mice, has potential for the therapeutic development of substrate-specific MALT1 inhibitors (examples for other proteases exist in the literature).

1st Revision - Editorial Decision 17th Apr 2026

Dear Prof. Beyaert,

Thank you for the submission of your revised manuscript to our editorial offices. I have now received the reports from the two referees that were asked to re-evaluate the study, you will find below. As you will see, the referees now fully support the publication of the study in EMBO reports.

Before we can proceed with formal acceptance, I have the editorial requests below I ask you to address in a final revised manuscript. Please also provide a final p-b-p-response to the editorial requests.

Editorial requests:

- Please shorten the title to not more than 100 characters (including spaces).

- Please order the manuscript sections like this, using only these names:
Title page - Abstract - Keywords - Introduction - Results - Discussion - Methods - Data availability section - Acknowledgements - Disclosure and Competing Interests Statement - References - Figure legends - Expanded View Figure legends

Please remove the running title and the list of abbreviations from the manuscript text file. Please define each abbreviation upon first mention in the text.

- Please make sure that the number "n" for how many independent experiments were performed, their nature (biological versus technical replicates), the bars and error bars (e.g. SEM, SD) and the test used to calculate p-values is indicated in the respective figure legends (main and EV figures). Please also check that all the p-values are explained in the legend, and that these fit to those shown in the figure. Please provide statistical testing where applicable. Please avoid the phrase 'independent experiment' but clearly state if these were biological or technical replicates. Please also indicate (e.g. with n.s.) if testing was performed, but the differences are not significant. In case n=2, please show the data as separate datapoints without error bars and statistics. See also:

https://link.springer.com/journal/44319/submission-guidelines#cms-Figure-and-data-presentation

If n<5, please show single datapoints for diagrams. Presently, most diagrams lack statistics or the 'n.s.'. Please provide proper and complete statistical testing for all diagrams shown. Moreover:
- Please note that the exact p values are not provided in the legend of figure 5A
- Please indicate the statistical test used for data analysis in the legend of figure 6A
- Please note that the box plots need to be defined in terms of minima, maxima, centre, bounds of box and whiskers, and percentile in the legends of figures 6A, B

- Please add the name of the database and the accession ID of the dataset to the data availability section.

- Please use our reference format (not numerical; et al needs to be used after 10 author names):
https://link.springer.com/journal/44319/submission-guidelines#cms-Reference-guidelines
- Please confirm that for all Western blot panels the lanes shown (including the loading control) were run on the same gel as the other proteins detected. I am not sure this is always the case. Please note that we discourage comparisons between samples on different gels/blots, even if the samples derive from one experiment, as confounding factors reduce comparability. If unavoidable, the figure legend must state that the samples derive from the same experiment and that gels/blots were processed in parallel. If a 'representative' loading control is shown for multiple gels/blots, the intra-gel controls should be shown in the source data files, and the figure legends should describe the data displayed accurately. See our author guidelines:

https://link.springer.com/journal/44319/submission-guidelines#cms-Figure-and-data-presentation (section 'Electrophoretic gels and blots').

- Thanks for uploading the source data (SD). The SD for Fig. EV is too large or can't be properly downloaded (or presented online) as is. Please upload only the numerical data with your final submission. Please consider using an external repository (like BioStudies) for the microscopic SD.

In addition, I would need from you uploaded separately:
- a short, two-sentence summary of the manuscript (not more than 35 words).
- two to four short (!) bullet points highlighting the key findings of your study (two lines each).
- a schematic summary figure as separate file that provides a sketch of the major findings (not a data image) in jpeg or tiff format (with the exact width of 550 pixels and the exact height of 300 pixels) that can be used as a visual synopsis on our website.

I look forward to seeing the further revised version of your manuscript when it is ready. Please let me know if you have questions regarding the revision.

Best,

Achim Breiling
Senior Editor
EMBO Reports

Referee #1:

The authors have appropriately addressed all major and minor concerns that we had previously raised

Referee #2:

The authors have responded to all of my comments to my satisfaction.

The authors have addressed all minor editorial requests.

2nd Revision - Editorial Decision 21st May 2026

Prof. Rudi Beyaert
Ghent University/VIB
Center for Inflammation Research
Technologiepark 71
Ghent 9052
Belgium

Dear Prof. Beyaert,

I am very pleased to accept your manuscript for publication in the next available issue of EMBO reports. Thank you for your contribution to our journal.

Your manuscript will be processed for publication by EMBO Press. It will be copy edited and you will receive page proofs prior to publication. Please note that you will be contacted by Springer Nature Author Services to complete licensing and payment information.

You may qualify for financial assistance for your publication charges - either via a Springer Nature fully open access agreement or an EMBO initiative. Check your eligibility: https://link.springer.com/journal/44319/how-to-publish-with-us

Should you be planning a Press Release on your article, please notify the production team via this form in order to coordinate publication and release dates: https://forms.cloud.microsoft/e/eH4A8JVP6g

If you have any questions, please do not hesitate to contact the Editorial Office. Thank you for your contribution to EMBO Reports.

Yours sincerely,

Achim Breiling
Senior Editor
EMBO Reports

>>> Please note that it is EMBO Reports policy for the transcript of the editorial process (containing referee reports and your response letter) to be published as an online supplement to each paper. If you do NOT want this, you will need to inform the Editorial Office via email immediately. More information is available here: https://link.springer.com/partners/embo-press/editorial-policies#Peer%20review

EMBO Press Author Checklist

Corresponding Author Name: Rudi Beyaert	
Journal Submitted to: EMBO reports	
Manuscript Number: EMBOR-2025-63159V1	

USEFUL LINKS FOR COMPLETING THIS FORM
The EMBO Journal - Author Guidelines
EMBO Reports - Author Guidelines
Molecular Systems Biology - Author Guidelines
EMBO Molecular Medicine - Author Guidelines

Reporting Checklist for Life Science Articles (updated January
This checklist is adapted from Materials Design Analysis Reporting (MDAR) Checklist for Authors. MDAR establishes a minimum set of requirements in transparent reporting in the life sciences (see Statement of Task: 10.31222/osf.io/9sm4x). Please follow the journal's guidelines in preparing your
Please note that a copy of this checklist will be published alongside your article.

Abridged guidelines for figures
1. Data
The data shown in figures should satisfy the following conditions:
- the data were obtained and processed according to the field's best practice and are presented to reflect the results of the experiments in an accurate and unbiased manner.
- ideally, figure panels should include only measurements that are directly comparable to each other and obtained with the same assay.
- plots include clearly labeled error bars for independent experiments and sample sizes. Unless justified, error bars should not be shown for technical
- if n<5, the individual data points from each experiment should be plotted. Any statistical test employed should be justified.
- Source Data should be included to report the data underlying figures according to the guidelines set out in the authorship guidelines on Data

2. Captions
Each figure caption should contain the following information, for each panel where they are relevant:
- a specification of the experimental system investigated (eg cell line, species name).
- the assay(s) and method(s) used to carry out the reported observations and measurements.
- an explicit mention of the biological and chemical entity(ies) that are being measured.
- an explicit mention of the biological and chemical entity(ies) that are altered/varied/perturbed in a controlled manner.
- the exact sample size (n) for each experimental group/condition, given as a number, not a range;
- a description of the sample collection allowing the reader to understand whether the samples represent technical or biological replicates (including how many animals, litters, cultures, etc.).
- a statement of how many times the experiment shown was independently replicated in the laboratory.
- definitions of statistical methods and measures:
  - common tests, such as t-test (please specify whether paired vs. unpaired), simple χ2 tests, Wilcoxon and Mann-Whitney tests, can be unambiguously identified by name only, but more complex techniques should be described in the methods section;
  - are tests one-sided or two-sided?
  - are there adjustments for multiple comparisons?
  - exact statistical test results, e.g., P values = x but not P values < x;
  - definition of 'center values' as median or average;
  - definition of error bars as s.d. or s.e.m.

Please complete ALL of the questions below.
Select "Not Applicable" only when the requested information is not relevant for your study.

Materials

Newly Created Materials	Information included in the manuscript?	In which section is the information available? (Reagents and Tools Table, Materials and Methods, Figures, Data Availability Section)
New materials and reagents need to be available; do any restrictions apply?	Not Applicable	

Antibodies	Information included in the manuscript?	In which section is the information available? (Reagents and Tools Table, Materials and Methods, Figures, Data Availability Section)
For **antibodies** provide the following information:<br>- Commercial antibodies: RRID (if possible) or supplier name, catalogue number and or/clone number<br>- Non-commercial: RRID or citation	Yes	Reagents and Tools Table and Material and Methods

DNA and RNA sequences	Information included in the manuscript?	In which section is the information available? (Reagents and Tools Table, Materials and Methods, Figures, Data Availability Section)
**Short novel DNA or RNA including primers, probes**: provide the sequences.	Yes	Reagents and Tools Table and Material and Methods

Cell materials	Information included in the manuscript?	In which section is the information available? (Reagents and Tools Table, Materials and Methods, Figures, Data Availability Section)
**Cell lines:** Provide species information, strain. Provide accession number in repository **OR** supplier name, catalog number, clone number, and/**OR** RRID.	Not Applicable	
**Primary cultures:** Provide species, strain, sex of origin, genetic modification status.	Yes	Material and Methods
Report if the cell lines were recently **authenticated** (e.g., by STR profiling) and tested for mycoplasma contamination.	Not Applicable	

Experimental animals	Information included in the manuscript?	In which section is the information available? (Reagents and Tools Table, Materials and Methods, Figures, Data Availability Section)
**Laboratory animals or Model organisms:** Provide species, strain, sex, age, genetic modification status. Provide accession number in repository **OR** supplier name, catalog number, clone number, **OR** RRID.	Yes	Reagents and Tools Table and Material and Methods
**Animal observed in or captured from the field:** Provide species, sex, and age where possible.	Yes	Figure legends and Material and Methods
Please detail **housing and husbandry conditions**.	Yes	Materila and Methods

Plants and microbes	Information included in the manuscript?	In which section is the information available? (Reagents and Tools Table, Materials and Methods, Figures, Data Availability Section)
**Plants:** provide species and strain, ecotype and cultivar where relevant, unique accession number if available, and source (including location for collected wild specimens).	Not Applicable	
**Microbes:** provide species and strain, unique accession number if available, and source.	Not Applicable	

Human research participants	Information included in the manuscript?	In which section is the information available? (Reagents and Tools Table, Materials and Methods, Figures, Data Availability Section)
If collected and within the bounds of privacy constraints report on age, sex and gender or ethnicity for all study participants.	Not Applicable	

Core facilities	Information included in the manuscript?	In which section is the information available? (Reagents and Tools Table, Materials and Methods, Figures, Data Availability Section)
If your work benefited from core facilities, was their service mentioned in the acknowledgments section?	Yes	Acknowledgments

Design

Study protocol	Information included in the manuscript?	In which section is the information available? (Reagents and Tools Table, Materials and Methods, Figures, Data Availability Section)
If study protocol has been **pre-registered, provide DOI in the manuscript**. For clinical trials, provide the trial registration number **OR** cite DOI.	Not Applicable	
Report the **clinical trial registration number** (at ClinicalTrials.gov or equivalent), where applicable.	Not Applicable	

Laboratory protocol	Information included in the manuscript?	In which section is the information available? (Reagents and Tools Table, Materials and Methods, Figures, Data Availability Section)
Provide DOI OR other citation details if **external detailed step-by-step protocols** are available.	Yes	Material and Methods

Experimental study design and statistics	Information included in the manuscript?	In which section is the information available? (Reagents and Tools Table, Materials and Methods, Figures, Data Availability Section)
Include a statement about **sample size** estimate even if no statistical methods were used.	Yes	Figure legends
Were any steps taken to minimize the effects of subjective bias when allocating animals/samples to treatment (e.g. **randomization procedure**)? If yes, have they been described?	Yes	Material and Methods
Include a statement about **blinding** even if no blinding was done.	Yes	Material and Methods
Describe **inclusion/exclusion criteria** if samples or animals were excluded from the analysis. Were the criteria pre-established?<br><br>If sample or data points were omitted from analysis, report if this was due to attrition or intentional exclusion and provide justification.	Yes	Material and Methods
For every figure, are **statistical tests** justified as appropriate? Do the data meet the assumptions of the tests (e.g., normal distribution)? Describe any methods used to assess it. Is there an estimate of variation within each group of data? Is the variance similar between the groups that are being statistically compared?	Yes	Figure legends

Sample definition and in-laboratory replication	Information included in the manuscript?	In which section is the information available? (Reagents and Tools Table, Materials and Methods, Figures, Data Availability Section)
In the figure legends: state number of times the experiment was **replicated** in laboratory.	Yes	Figure legends
In the figure legends: define whether data describe **technical or biological replicates**.	Yes	Figure legends

Ethics

Ethics	Information included in the manuscript?	In which section is the information available? (Reagents and Tools Table, Materials and Methods, Figures, Data Availability Section)
Studies involving **human participants**: State details of **authority granting ethics approval** (IRB or equivalent committee(s), provide reference number for approval.	Not Applicable	
Studies involving **human participants**: Include a statement confirming that **informed consent** was obtained from all subjects and that the experiments conformed to the principles set out in the WMA Declaration of Helsinki and the Department of Health and Human Services Belmont Report.	Not Applicable	
Studies involving **human participants:** For publication of **patient photos**, include a statement confirming that consent to publish was obtained.	Not Applicable	
Studies involving experimental **animals**: State details of **authority granting ethics approval** (IRB or equivalent committee(s), provide reference number for approval. Include a statement of compliance with ethical regulations.	Yes	Material and Methods
Studies involving **specimen and field samples:** State if relevant **permits** obtained, provide details of authority approving study; if none were required, explain why.	Not Applicable	

Dual Use Research of Concern (DURC)	Information included in the manuscript?	In which section is the information available? (Reagents and Tools Table, Materials and Methods, Figures, Data Availability Section)
Could your study fall under dual use research restrictions? Please check biosecurity documents and list of **select agents and toxins** (CDC): https://www.selectagents.gov/sat/list.htm	Not Applicable	
If you used a select agent, is the security level of the lab appropriate and reported in the manuscript?	Not Applicable	
If a study is subject to dual use research of concern regulations, is the name of the **authority granting approval and reference number** for the regulatory approval provided in the manuscript?	Not Applicable	

Reporting

The MDAR framework recommends adoption of discipline-specific guidelines, established and endorsed through community initiatives. Journals have their own policy about requiring specific guidelines and recommendations to complement MDAR.

Adherence to community standards	Information included in the manuscript?	In which section is the information available? (Reagents and Tools Table, Materials and Methods, Figures, Data Availability Section)
State if relevant guidelines or checklists (e.g., **ICMJE, MIBBI, ARRIVE, PRISMA**) have been followed or provided.	Not Applicable	
For **tumor marker prognostic studies,** we recommend that you follow the **REMARK** reporting guidelines (see link list at top right). See author guidelines, under 'Reporting Guidelines'. Please confirm you have followed these guidelines.	Not Applicable	
For **phase II and III randomized controlled trials**, please refer to the **CONSORT** flow diagram (see link list at top right) and submit the CONSORT checklist (see link list at top right) with your submission. See author guidelines, under 'Reporting Guidelines'. Please confirm you have submitted this list.	Not Applicable	

Data Availability

Data availability	Information included in the manuscript?	In which section is the information available? (Reagents and Tools Table, Materials and Methods, Figures, Data Availability Section)
Have **primary datasets** been deposited according to the journal's guidelines (see 'Data Deposition' section) and the respective accession numbers provided in the Data Availability Section?	Yes	Material and Methods / Data availability
Were **human clinical and genomic datasets** deposited in a public access-controlled repository in accordance to ethical obligations to the patients and to the applicable consent agreement?	Not Applicable	
Are **computational models** that are central and integral to a study available without restrictions in a machine-readable form? Were the relevant accession numbers or links provided?	Not Applicable	
If publicly available data were reused, provide the respective **data citations in the reference list**.	Not Applicable	
